# A novel approach for the prediction of species-specific biotransformation of xenobiotic/drug molecules by the human gut microbiota

**DOI:** 10.1038/s41598-017-10203-6

**Published:** 2017-08-29

**Authors:** Ashok K. Sharma, Shubham K. Jaiswal, Nikhil Chaudhary, Vineet K. Sharma

**Affiliations:** Metagenomics and Systems Biology Laboratory, Indian Institute of Science Education and Research, Bhopal, Madhya Pradesh India

## Abstract

The human gut microbiota is constituted of a diverse group of microbial species harbouring an enormous metabolic potential, which can alter the metabolism of orally administered drugs leading to individual/population-specific differences in drug responses. Considering the large heterogeneous pool of human gut bacteria and their metabolic enzymes, investigation of species-specific contribution to xenobiotic/drug metabolism by experimental studies is a challenging task. Therefore, we have developed a novel computational approach to predict the metabolic enzymes and gut bacterial species, which can potentially carry out the biotransformation of a xenobiotic/drug molecule. A substrate database was constructed for metabolic enzymes from 491 available human gut bacteria. The structural properties (fingerprints) from these substrates were extracted and used for the development of random forest models, which displayed average accuracies of up to 98.61% and 93.25% on cross-validation and blind set, respectively. After the prediction of EC subclass, the specific metabolic enzyme (EC) is identified using a molecular similarity search. The performance was further evaluated on an independent set of FDA-approved drugs and other clinically important molecules. To our knowledge, this is the only available approach implemented as ‘DrugBug’ tool for the prediction of xenobiotic/drug metabolism by metabolic enzymes of human gut microbiota.

## Introduction

The human gut harbours more than 100 trillion microbial cells belonging to about 1,000 different bacterial species, and hence, it constitutes a huge reservoir of metabolic enzymes in the gut capable of showing a vast array of metabolic activities in addition to those carried out by the host enzymes^1^. These bacterial metabolic activities affect human metabolism, physiology, nutrition uptake and immune system activities, and thus have significant implications for human health and diseases such as inflammatory bowel disease, obesity, and Type II diabetes^2–4^. The diverse metabolic activities of gut microbes can modulate the host metabolic machinery by interfering with the processes of energy harvesting and extraction of essential nutrients from dietary food, and through the metabolism of xenobiotic/drug molecules^5–12^.

Reports on xenobiotic/drug metabolism by gut bacteria have been known since last three decades. However, the effect of gut microbiota on metabolism, bioavailability, bioactivity, and toxicity of xenobiotic/drug molecules is yet underexplored. Furthermore, the structure of gut microbiota in any individual is primarily shaped by environmental factors such as diet, geography, antibiotics, and probiotics as well by genetic factors like minor genomic variations of host genome^13^. Thus, the metabolism of any xenobiotic/drug is likely to be influenced by individual and population-specific variations of the gut metagenome along with host-mediated metabolism.

Several drugs such as acetaminophen and digoxin have shown population-specific variations in drug response which correlates with the metabolic activities of bacteria in the human gut^14–17^. Similarly, at least 40 therapeutic drugs have been reported to be metabolized by the gut microbes in Pharmacomicrobiomics database. However, except for a few cases, the microbial species and metabolic enzymes are still uncharacterized^18, 19^. A few cases that demonstrate the metabolism of a drug molecule has been shown to be carried out by a gut bacterial species are metabolism of chloramphenicol by *Escherichia coli* ^20^, sorivudine by *Bacteroides eggerthii* and *Bacteroides vulgatus* ^21^, cyclophosphamide by Firmicutes^22^ and olaquindox by *Escherichia coli* ^23^.

The experimental methods of metabolic profiling such as Nuclear Magnetic Resonance (NMR) spectroscopy and Liquid Chromatography-Mass Spectrometry (LC-MS), can be utilized to determine the corresponding metabolic enzymes and bacteria responsible for the biotransformation of xenobiotic/drug molecule. However, the complex and dynamic metabolic interactions between host-bacteria and bacteria-bacteria have largely impeded the experimental determination of the species-specific contribution of gut microbes in the metabolism of xenobiotic/drug molecules. It is further limited by the time-consuming and tedious nature of experimental studies, which involve deep metabolic profiling of host gut microbiota for each xenobiotic/drug molecule. Therefore, for most of the orally administered drugs that encounter gut microbiota before their absorption, the gut microbial species and the corresponding enzymes capable of their metabolism are largely unknown. In this scenario, an efficient computational method is required for the prediction of microbial species and enzymes, which could potentially metabolize a xenobiotic/drug in the human gut.

Presently, a few tools which are available for predicting drug metabolism are primarily based on human phase-I (hydrolysis, oxidation and reduction reactions) and phase-II (conjugation reactions) metabolic processes, namely MetaSite^24^, Metaprint2D^25^, ADMET predictor, Metabolism Module simulations Plus (http://www.simulations-plus.com/), RS-WebPredictor^26^ and FAME^27^. To our knowledge, there is no tool or computational approach available to predict the biotransformation of xenobiotic/drug in human gut by the metabolic enzymes encoded by the gut bacteria. An enzyme is capable of acting on molecules which are structurally similar to their substrate, and this property is known as promiscuity. Therefore, the molecular properties of substrates of all known metabolic enzymes encoded by the gut microbiota can be used to predict the metabolic enzymes and gut bacteria which can potentially carry out the biotransformation of xenobiotic/drug molecules^28–31^. Therefore, in this work, we report a novel methodology developed by integrating chemoinformatics and machine learning methods for the prediction of the metabolic enzyme and the corresponding bacterial species capable of metabolizing a given xenobiotic/drug molecule at the first/initial step.

## Results

### Metabolic enzymes and substrate databases

To develop an approach for the prediction of metabolic enzymes and gut bacteria, which can potentially act on a xenobiotic/drug molecule, the first step is the construction of a comprehensive dataset of metabolic enzymes and their substrates from all known human gut bacteria. Therefore, a database of metabolic enzymes was constructed from 491 human gut bacterial genomes, which contained 324,697 metabolic enzymes assigned with EC numbers. For these metabolic enzymes belonging to different EC classes, the substrate database was constructed containing a total of 1,609 molecules (Figure S1a). Using this approach, we could identify the substrate molecules for metabolic enzymes of gut bacteria. These substrate molecules were utilized for constructing the training dataset for machine learning methods.

The distribution of substrates for enzymes present in different EC classes revealed an imbalance in their respective numbers in the different classes. The number of molecules metabolized by enzymes from the first two EC (EC1 and EC2) classes was the highest (65.75%), whereas enzymes from EC5 and EC6 classes are known to metabolize only 6.83% of the total number of molecules. The enzymes in EC1 and EC2 classes are oxidoreductases and transferases, respectively, which represent the common metabolic reactions in the human gut. Thus, a large number of substrate molecules are metabolized through these reactions. On the other hand, the enzymatic reactions in EC5 (isomerases) and EC6 (ligases) classes are less common, and hence, a lower number of substrate molecules are known to be metabolized by these classes. Similarly, the distribution of molecules for enzymes in different EC subclasses shows that out of 55 subclasses, 22 subclasses can metabolize less than five substrate molecules, whereas only three subclasses can metabolize more than 100 molecules (Figure S1b-g). The above analysis points towards ‘class imbalance’ as a result of the disparity in the number of molecules (instances) metabolized by different EC classes.

The performance of machine learning methods is known to be affected by class imbalance and thus, requires a considerable number of examples/instances during training for reliable prediction^32^. Therefore, to resolve the class imbalance problem upsampling strategy was employed using the upSample function of the ‘Caret Package’ in R (Text S1). The downsampling was also performed but showed poor performance in comparison to upsampling and without-upsampling, and hence, was not considered for subsequent analysis in this study (Text S1). The final models were prepared using both datasets, i.e. with-upsampling and without-upsampling.

### Fingerprints generated for each molecule

The structural features of a substrate molecule can be represented through substructure-based fingerprints, which could be used as an input feature for constructing machine learning-based models or for performing molecular similarity search. Thus, for each molecule in the substrate database, 10 standard fingerprints were calculated using ‘PaDEL’^33^. The best attributes or bits from each fingerprint were selected and combined to create a new hybrid fingerprint (219 bits). In this case, the important attributes are the ones which can discriminate between the different EC classes and are also unrelated to each other. The detailed description of contribution from each fingerprint to the hybrid fingerprint is provided in Supplementary Table S1.

### Diversity of substrate molecules in different EC classes and subclasses

**Figure 1:**
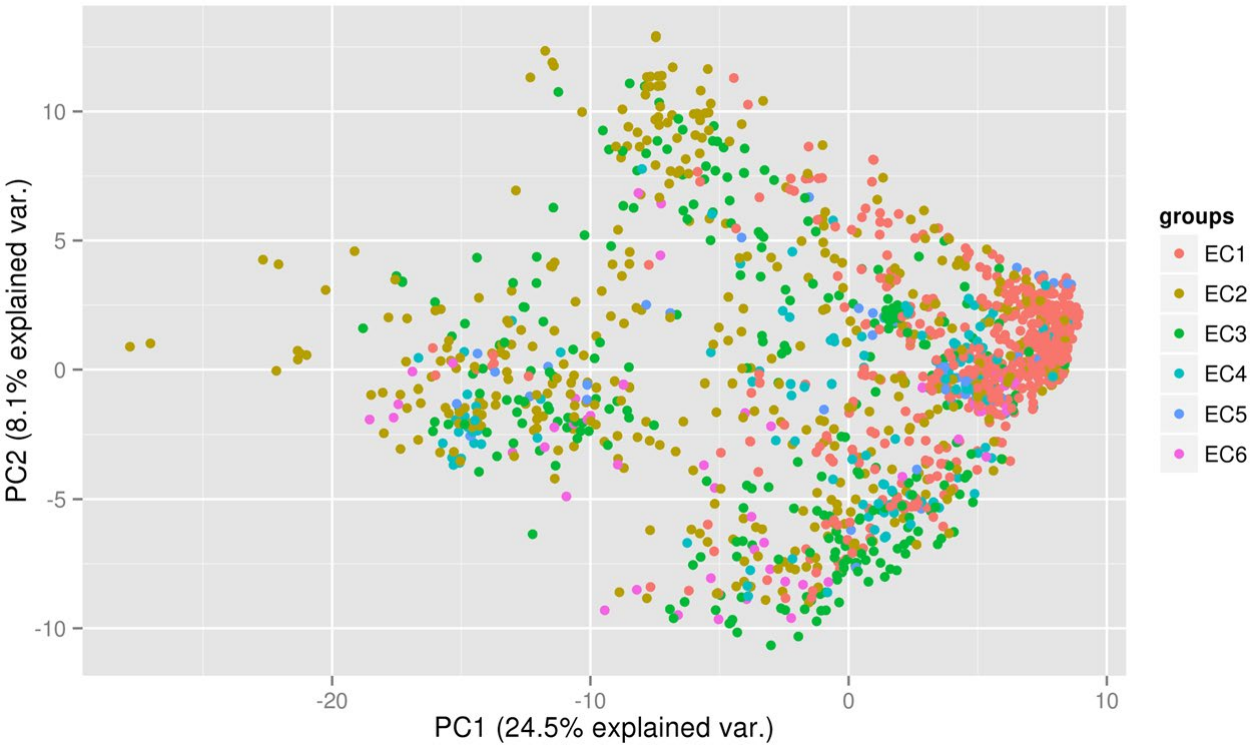
The distribution of substrate molecules into the six EC classes is shown by Principal Component Analysis. Each substrate molecule of a respective class is represented by colour coded circles.

To identify the components which show the highest variance among the six EC classes and subclasses of each EC class, Principal Component Analysis (PCA) was performed on all 1,609 molecules present in the substrate database using the hybrid fingerprint. For the six EC classes taken together, the variance is observed to decrease significantly from PC-1 to PC-6 (Figure S2a). Similarly, for all EC subclasses belonging to an EC class, the variance showed a significant decrease from PC-1 to PC-10, and the same trend was observed for all the six EC classes (Figure S2b). The variance between PC-1 and PC-2 for the six EC classes was 24.5% and 8.1%, respectively (Fig. 1). Similarly, the variance between PC-1 and PC-2 for the EC subclasses belonging to an EC class is as follows: EC1: PC-1 = 19.2% and PC-2 = 7.8%, EC2: PC-1 = 22.6% and PC-2 = 9.8%, EC3: PC-1 = 18.1% and PC-2 = 12.0%, EC4: PC-1 = 28.7% and PC-2 = 7.6%, EC5: PC-1 = 33.4% and PC-2 = 11.3%, EC6: PC-1 = 29.0% and PC-2 = 12.4% (Figure S3a–f). The results of PCA analysis indicate that the dataset is highly diverse for developing a prediction model, and since none of the principal component pairs add up to 50% of the variation, only a very limited amount of variable reduction can be done. Therefore, a robust machine learning method is required to develop reliable classification models.

### EC class and subclass specific random forest (RF) models were constructed for classification

To select the best performing model for classification, the performances of different machine learning approaches were compared using Weka^34^. For this evaluation (using 10-fold cross-validation), the complete dataset of molecules belonging to the six different EC classes was used as the input to calculate the percentage of correctly classified instances for all the six EC classes. Among the different machine learning approaches, random forest (RF) showed the best performance (Supplementary Table S2, Methods), and hence, was considered for further optimization using the randomForest package in R to achieve the lowest %OOB (Out of Bag) error and the highest classification accuracy. The optimization of parameters such as mtry, which is a subset of variables randomly selected at each node for the classification, and ntree, which is the number of classification trees (independent models) in the forest, was carried out for each fingerprint. To choose the best fingerprint for constructing the RF model, the mtry values were optimized for each fingerprint using the tuneRF function of random forest package in R. The tuneRF function looks for the best mtry value with the lowest %OOB error in a range of mtry values around the specified mtry value, with a defined step size. The specified mtry values were calculated as the square root of the total number of bits (predictor variables) used for a given fingerprint. The ‘stepFactor’ (for step size) and ‘improve’ functions were kept constant for all the mtry optimizations, and were 2 and 0.05, respectively.

**Figure 2:**
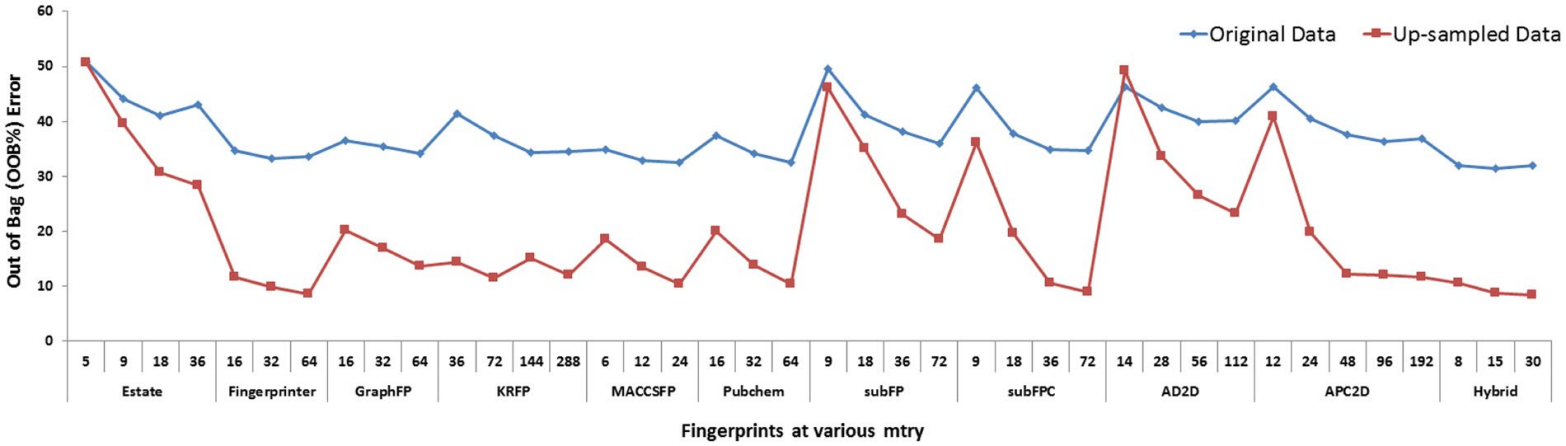
Optimization of parameters to construct the final RF model for classification into six EC classes.

For each fingerprint, the %OOB (Out of Bag) error values were calculated at the optimized mtry values and at the ntree value of 500. This ntree value was selected based on the saturation of %OOB value (Supplementary Table S3). The above analysis was carried out for each fingerprint using both without-upsampling and with–upsampling training datasets. In the case of six EC classes, among all the 10 fingerprints and hybrid fingerprint, the lowest (8.42) %OOB error was shown by the hybrid fingerprint (Fig. 2). It was also noted that the model prepared using with–upsampling dataset displayed better performance for all fingerprints as compared to the model prepared using without-upsampling dataset. RF models for each EC class prepared using up-sampled data were optimized separately to achieve high accuracy for classification into their respective EC subclass. Similarly, for EC1, EC2, EC3, EC4, EC5 and EC6, the hybrid fingerprint displayed the lowest %OOB error of 2.97, 13.23, 2.96, 7.77, 3.75 and 1.11, respectively (Figure S4). Based on the %OOB error, the three parameters (mtry, ntree and best fingerprint) were selected for constructing RF models for EC classes and subclasses (Fig. 2 and Figure S4). The finalized RF models constructed using hybrid fingerprint at optimized mtry and ntree were used for further validations.

### RF models showed high classification accuracy on different validation datasets

EC class-specific and subclass-specific RF models were constructed using hybrid fingerprint using both with-upsampling and without-upsampling datasets and were considered for the evaluation. The performance evaluation of these hybrid RF models was carried out using 10-fold cross-validation, splitting and testing, and blind set. For EC class, the RF model with-upsampling dataset displayed higher accuracy of 97.19, 95.75 and 91.18 and MCC values of 0.89, 0.84 and 0.59 for 10-fold cross-validation, splitting and testing, and blind set, respectively, as compared to RF model without-upsampling dataset (Table 1). Similarly, for the EC subclasses, the RF model with-upsampling displayed higher (80.83–100%) accuracy as well as higher (0.67–1) MCC values for 10-fold cross-validation, splitting and testing, and blind set as compared to RF model without-upsampling dataset (Table 2). It is apparent that the MCC values on blind set were lower only in the case of EC class-specific RF module (Table 1), whereas in the cases of all individual EC classes (Table 2), it was similar to the ten-fold cross-validation and splitting and testing sets. Considering the complex and heterogeneous nature of the data (also supported by the PCA analysis, Fig. 1), the reported MCC values were the maximum which could be achieved on the available data.

**Table 1:** Performance evaluation of EC class-specific RF model using three different methods.

Validation on test sets	Hybrid Fingerprint									
	RF model without-upsampling dataset					RF model with-upsampling dataset				
	TPR (%)	TNR (%)	PPV (%)	ACC (%)	MCC	TPR (%)	TNR (%)	PPV (%)	ACC (%)	MCC
CV-10 FOLD	52.83	92.63	60.32	89.33	0.49	91.58	98.32	91.46	97.19	0.89
Splitting and Testing	60.84	92.71	49.05	88.94	0.46	87.02	97.49	87.25	95.75	0.84
Blind Set	55.52	94.17	66.15	92.14	0.54	67.41	94	63.19	91.18	0.59

TPR = True Positive Rate or Sensitivity, TNR = True Negative Rate or Specificity, PPV = Positive Predictive Value or Precision, ACC = Accuracy, MCC = Matthews correlation coefficient.

**Table 2:** Performance evaluation of EC subclass-specific RF models using three different methods.

Validation on test sets		Hybrid Fingerprint									
		RF model without-upsampling dataset					RF model with-upsampling dataset				
		TPR (%)	TNR (%)	PPV (%)	ACC (%)	MCC	TPR (%)	TNR (%)	PPV (%)	ACC (%)	MCC
EC1	CV-10 FOLD	62.41	97.1	65.11	95.9	0.61	97.03	99.82	97.17	99.67	0.97
	Splitting and Testing	82.14	97.02	75.71	95.26	0.75	97.09	99.82	97.02	99.66	0.97
	Blind Set	87.62	98.18	95.07	97.5	0.89	83.17	98.73	83.67	97.3	0.81
EC2	CV-10 FOLD	55.65	93.39	56.96	89.27	0.50	86.67	98.52	86.26	97.33	0.85
	Splitting and Testing	66.94	94.23	64.75	90.34	0.6	85.36	98.44	85.83	97.17	0.84
	Blind Set	81.76	94.36	81.75	91.02	0.76	83.09	96.12	80.95	92.86	0.77
EC3	CV-10 FOLD	65.38	97.38	78.82	96.3	0.68	97.04	99.63	97	99.34	0.97
	Splitting and Testing	88.77	96.56	93.91	93.93	0.87	95.4	99.42	95.3	98.96	0.95
	Blind Set	95	97.5	94.44	96.3	0.92	95	97.5	94.44	96.3	0.92
EC4	CV-10 FOLD	59.21	88.86	69.42	86.27	0.53	91.86	98.84	91.47	97.96	0.9
	Splitting and Testing	70	76.98	48.91	73.15	0.34	88.83	98.48	89.06	97.26	0.87
	Blind Set	78.57	82.5	80.55	83.33	0.63	80.83	91.22	75	86.54	0.67
EC5*	CV-10 FOLD	76.67	83.54	85	91.16	0.7	95.62	98.91	95.93	98.25	0.95
	Splitting and Testing	88.89	75	93.75	86.36	0.62	97.77	99.39	97.5	99	0.97
EC6*	CV-10 FOLD	95.74	93.77	92.61	95.16	0.89	97.78	99.44	97.79	99.11	0.97
	Splitting and Testing	95	95	90	92.86	0.85	98	99.46	97.78	99.11	0.97

*For EC5 and EC6 classes, the validation could not be performed on blind set due to less representation of molecules in these classes. The average accuracy of cross-validation, splitting and testing and the blind set was 98.61, 98.52 and 93.25%, respectively.

TPR = True Positive Rate or Sensitivity, TNR = True Negative Rate or Specificity, PPV = Positive Predictive Value or Precision, ACC = Accuracy, MCC = Matthews correlation coefficient.

Additionally, the performance of EC1 and EC2 subclass-specific RF models was relatively better than EC5 and EC6 RF models due to the larger number of molecules available for training in the former classes as compared to the latter classes. In future, with the availability of more substrate molecules for EC5 and EC6 classes, the prediction by corresponding models can be improved. The performance of RF models on different validation sets attests the strength of this approach in identifying the EC class and subclass capable of biotransforming the substrate molecule. Since the best performance (Tables 1 and 2) was shown by the RF models constructed using the hybrid fingerprint on with-upsampling dataset, the same has been used as the default in respective RF modules for the prediction on web server.

### Web server for the prediction of metabolic enzymes and gut bacteria

To facilitate the prediction of metabolic enzymes and the associated gut bacteria responsible for the biotransformation of any xenobiotic/drug molecule, we have developed a web server ‘DrugBug’ by implementing predictive RF modules along with the similarity search module.

#### Construction of predictive RF modules

Two different RF modules were constructed using best performing RF models and were included in DrugBug tool and web server (Text S2).

#### EC class-specific RF module (RF module 1)

**Figure 3:**
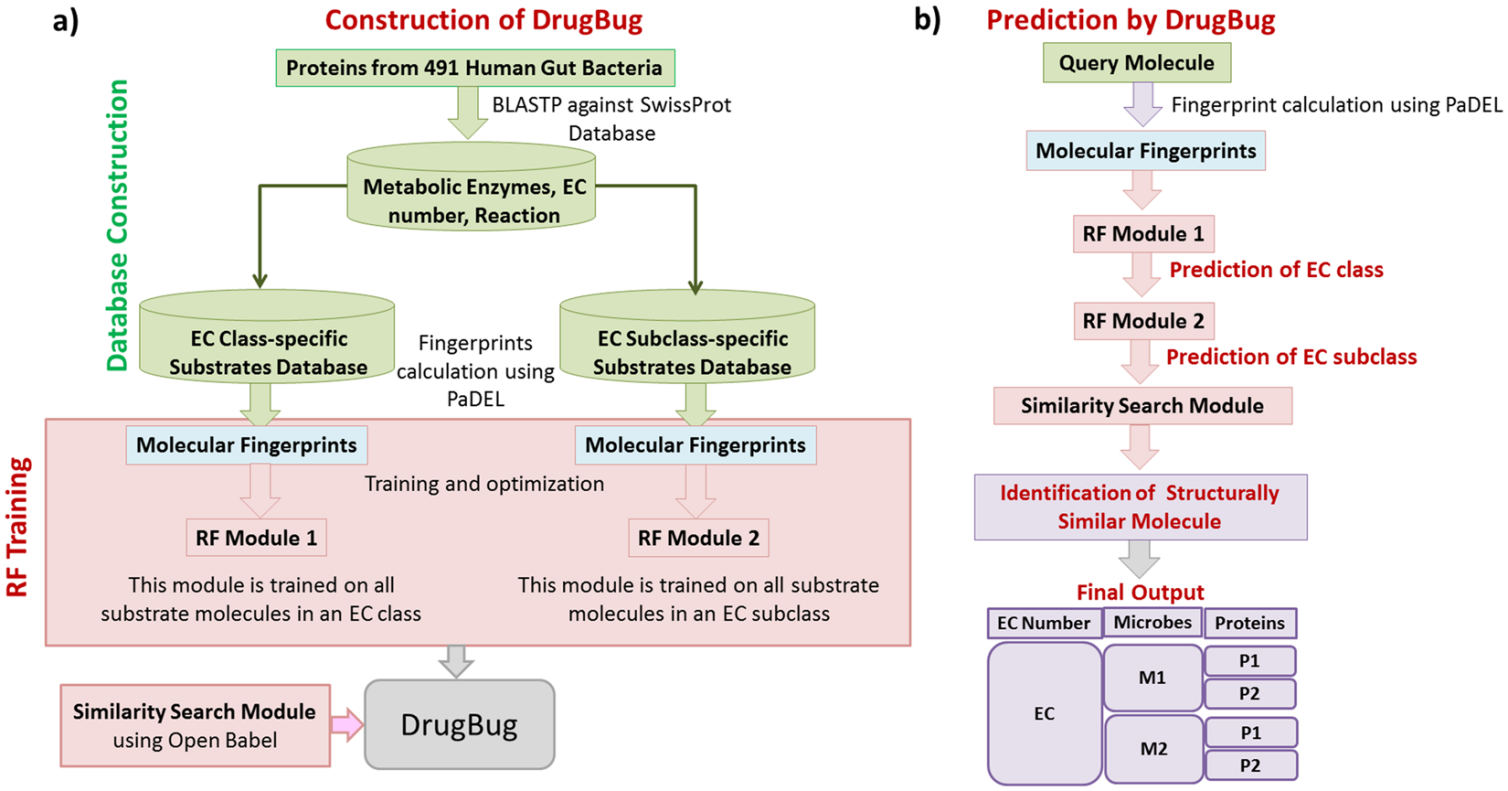
(**a**) Complete workflow for the construction of DrugBug. Figure 3 (**b**) Steps for the analysis of a query molecule through DrugBug web server. DrugBug consists of three different components namely, EC class-specific RF module (RF module 1), EC subclass-specific RF module (RF module 2) and a similarity search module. In the given example, the query molecule is analyzed by these modules to identify the EC number and the corresponding metabolic enzyme which was found in two bacterial genomes (M1 and M2). In each of the predicted bacteria (M1 and M2), two or more proteins (P1 and P2) similar to the EC enzyme were found.

This module was trained on fingerprints derived from all substrate molecules present in substrate database of all the six EC classes. This module predicts the EC class capable of carrying out the biotransformation of a query molecule (Fig. 3a).

#### EC subclass-specific module (RF module 2)

RF modules were constructed for each of the EC classes and were trained on fingerprints of substrate molecules belonging to a particular EC subclass. This module predicts the EC subclass capable of carrying out the biotransformation of a query molecule (Fig. 3a).

A three-step analysis is followed by the web server to predict the gut bacterial metabolic enzyme (EC) and the respective bacteria for the biotransformation of a query molecule. In the first step, the extracted features (fingerprints) from the query molecule are analyzed through the RF module 1 to predict the EC class (out of any of the six EC classes) for the input molecule. After determination of the EC class, in the second step, the same molecular features pass through the RF module 2 for identification of EC subclasses (two-digit EC class) of the respective EC class for the query molecule. In both steps, the user has the flexibility to choose from the available RF models, sampling methods, and also the prediction probability threshold values (Text S3). In the third step, a molecule that is structurally similar to the input molecule is identified using molecular similarity search performed using ‘Open Babel’ against the substrate database of the EC subclass predicted in the second step. Any of the four fingerprints FP2, FP4, MACCSFP and hybrid (combination of results from FP2, FP4 and MACCSFP) can be chosen for the similarity search calculations using Open Babel. A user can also use the tanimoto coefficient cut-off value and similarity search parameters such as Identity, E-value, and Q-coverage, to act as filters among the identified hits. On the basis of the significant hit, the exact four-digit EC number which corresponds to the enzyme capable of carrying out the first/initial step in the biotransformation of the query molecule, and the information about gut bacteria which harbour this enzyme is also provided. The complete flowchart showing the steps for prediction of the metabolic enzyme and the gut bacteria that can potentially carry out the biotransformation of a query molecule is shown in Fig. 3b.

In the case of a query drug/xenobiotic molecule, which can be biotransformed by multiple EC classes, the classifier will predict only a single EC class and sub-class after the first and second steps of prediction. In the predicted EC subclass, multiple enzymes belonging to the same subclass can be predicted through Open Babel structural similarity search. Therefore, if a molecule is a substrate for multiple enzymes belonging to the same EC sub-class, then the DrugBug approach will predict all the enzymes. However, it will not predict the enzymes from a different EC class or EC sub-class.

### Prediction of gut microbial enzymes for the biotransformation of known drugs using ‘DrugBug’

To assess the performance of DrugBug approach, FDA-approved drugs, and other clinically important molecules were used as the real dataset. DrugBug was used to predict the enzymes and gut bacteria harbouring these enzymes which could potentially metabolize these drug molecules. At present, the information about the metabolism of drugs by human gut bacteria is limited only to a few drug molecules. Some of these selected cases were analyzed using DrugBug, and the predictions were in agreement with their partially known biotransformation information (Table 3). Furthermore, for these cases, DrugBug could correctly predict the specific bacterial enzyme and the gut bacteria (with taxonomy) which could carry out their biotransformation. This is the first report for the prediction of gut bacteria and the metabolic enzymes for biotransformation of these 10 drugs. Considering the variations in gut microbiota due to population differences, age, gender, etc. the knowledge of gut-microbe-linked drug metabolism could help in predicting the individual-specific metabolism of a drug which is significant for pharmacological studies and personalized medicine.

**Table 3:** Prediction of gut bacteria and the corresponding metabolic enzyme for biotransformation of some selected FDA-approved drugs and other clinically important molecules.

**Drug**	**Previous Reports**	**DrugBug Prediction**
**Ginsenoside Rb1** ^2^	**Organism:** Human and Gut microbiota (Bacteroides and Bifidobacterium) **Type of reaction:** Hydrolysis **EC:** 3.2.1.192* **Enzyme:** Ginsenoside Rb1 beta-glucosidase* ref. 58	**Organism:** *Escherichia coli MS 175*-*1*, *Bacteroides sp*. *3 1 23*, *Citrobacter sp*. *30 2*, *Enterobacter cloacae subsp*. *Cloacae NCTC 9394*, *Bifidobacteium animalis subsp*. *Lactis*-*AD011* **Enzyme class:** Hydrolses **EC:** 3.21.21 **Enzyme:** Glycosyl hydrolase family 3, thermostable β-glucosidase B, periplasmic β-glucosidase.
**Quercetin-3-glucoside** ^2^	**Organism:** Human and Gut microbiota (Eubacterium and Enterococcus) **Type of reaction:** Deglycosylation **EC:** 2.4.1.239* **Enzyme:** flavonol-3-O-glucoside glucosyltransferase* ref. 59	**Organisms:** *Escherichia coli MS 187*-*1*, *Pseudomonas sp*. *2 1 26*, *Streptococcus sp*. *2 1 36FAA*, *Enterobacter cloacae subsp*. *Clocae NCTC 9394*, *Enterococcus faecium TX1330* **Enzyme class:** Transferases **EC:** 2.4.1.- **Enzyme:** Glucans biosynthesis glucosyltransferase H, rhamnosyltransferase 1 subunit A, Accessory Sec system glycosylation protein GtfA, Membrane glycosyltransferase, glycosyltransferase group 2 family protein
**LoperamideOxide** ^1^	**Organisms:** Human and Gut microbiota **Type of reaction:** Reduction **EC:** 1.14.13.97* **Enzyme:** taurochenodeoxycholate 6alpha-hydroxylase* ref^+^. 60	**Organisms**: *Escherichia coli MS 119*-*7*, *Citrobacter youngae ATCC 29220*, *Klebsiella sp*. *4 1 44FAA*, *Listeria innocua ATCC*, *Paenibacillus sp*. *HGF7* **Enzyme class**: Oxidoreductases **EC:** 1.14.-.-/1.14.13.- **Enzyme:** Rieske 2Fe-2S domain potein, putative monooxygenase MoxC, FMN-dependent oxidoreductase
**Methamphetamine** ^1^	**Organisms:** Human and Gut microbiota (Lactobacilli, Enterococci and Clostridia) **Type of reaction:** Oxidoreductase/ Demethylation **EC:** 1.14.11.-* and 1.14.13.-* **Enzyme:** Hydroxylases, monooxygenases, dioxygeases, demethylases ref. 61	**Organisms:** *Escherichia coli MS 196*-*1*, *Ralstonia sp*. *5 2 56FAA*, *Citrobacter freundii 4 7 47CFAA*, *Lactobacillus rhamnosus ATCC 21052*, *Enterococcus faecalis TX0104* **Enzyme class:** Oxidoreductases **EC:** 1.14.13.- and 1.14.14.1 **Enzyme:** 2-polyprenyl-6-methoxyphenol 4-hydroxylase, 2-nonaprenyl-3-methyl-6-methoxy-1,4-benzoquinol hydroxylase, 2-octaprenyl-3-methyl-6-methoxy-1,4-benzoquinol hydroxylase, pyridine nucleotide-disulfide oxidoreductase family protein
**Omeprazole** ^1^	**Organisms:** Human and Gut microbiota **Type of reaction:** Reduction **EC:** 1.14.13.48* and 1.14.13.49* **Enzyme:** (S)-limonene 6-monooxygenase* and (S)-limonene 7-monooxygenase* ref. 62	**Organisms:** *Bacillus sp*. *7 6 55CFAA CT2*, *Paenibacillus sp*. *HGF7*, *Paenibacillus sp*. *HGF5*, *Ralstonia sp*. *5 7 47FAA* **Enzyme class:** Oxidoreductase **EC:** 1.14.14.1 **Enzyme:** FAD binding domain protein, bifunctional P-450/NADPH-P450 reductase, FAD binding domain protein, Hypothetical protein (100% Identical with cytochrome P450 reductase of bacillus cereus)
**Sorivudine** ^**1**^	**Organism:** Human and Gut microbiota (Bacteroides) **Type of reaction:** Phosphotransferase **EC:** 2.7.1.21* **Enzyme:** thymidine kinase* ref. 21	**Organism**: *Escherichia coli MS 69*-*1*, *Lactobacillus reuteri MM2*-*3*, *Klebsiella sp*. *4 1 44FAA*, *Proteus mirabilis WGLW6*, *Bacteroides sp*. *3 1 23*, *Escherichia coli SE11* **Enzyme class:** *Transferases* **EC:** 2.7.1.48 **Enzyme:** uridine kinase, uridine/cytidine kinase
**Lactulose** ^1^	**Organism:** Human and Gut microbiota (Bacteroides, Bifidobacterium, clostridium and lactobacillus) **Type of reaction:** Hydrolysis **EC:** Unknown **Enzyme:** Unknown ref. 58	**Organism**: *Bifidobacterium longum subsp*. *longum 2*-*2B*, *Lactobacillus brevis subsp*. *gravesensis ATCC 27305*, *Megamonas hypermegale ART12 1*, *Clostridium leptum DSM 753* **Enzyme class:** Hydrolases **EC:** 3.2.1.185 **Enzyme:** Putative glycosylhydrolase
**Zonisamide** ^1^	**Organism:** Human and Gut microbiota **Type of reaction:** Reduction **EC:** 1.14.13.97* Enzyme: taurochenodeoxycholate 6alpha-hydroxylase* ref. 63	**Organism:** *Pseudomonas sp*. *2 1 26*, *Ralstonia sp*. *5 7 47FAA*, *Klebsiella sp*. *4 1 44FAA*, *Corynebacterium ammoniagenes DSM 20306* **Enzyme class:** Oxidoreductase **EC:** 1.14.12.- **Enzyme**: toluate 1,2-dioxygenase subunit beta, benzoate 1,2-dioxygenase, small subunit, benzoate 1,2-dioxygenase, large subunit
**Cycasin** ^**2**^	**Organism:** Human and Gut microbiota **Type of reaction:** Hydrolysis **EC:** 3.2.1.21* **Enzyme:** Beta-glucosidase* [Google book] ref. 64	**Organism:** *Klebsiella sp*. *MS 92*-*3*, *Escherichia sp*. *4 1 40B*, *Bacteroides ovatus ATCC 8483*, *Lactobacillus helveticus DSM 20075*, *Bifidobacterium adolescentis L2*-*32* **Enzyme class:** Hydrolases **EC:** 3.2.1.23 **Enzyme:** glycosyl hydrolase family 2, beta-galactosidase
**Cyadox** ^**3**^	**Organism:** Human and Gut microbiota **Type of reaction:** Reduction **EC:** 1.14.-.-* **Enzyme:** Catalase and cytochrome P450s* refs 65, 66	**Organism:** *Escherichia sp*. *4 1 40B*, *Citrobacter youngae ATCC 29220*, *Listeria innocua ATCC 33091*, *Paenibacillus sp*. *HGF7*, *Paenibacillus sp*. *HGF5* **Enzyme class:** Oxidoreductase **EC:** 1.14.-.- **Enzyme:** Putative dioxygenase subunit alpha yeaW, putative transporting ATPase, rieske 2Fe-2S domain protein, putative monooxygenase MoxC, FMN-dependent oxidoreductase, polyketide biosynthesis cytochrome P450 PksS

*Enzyme was known in human host

^1^FDA approved drug;^2^: Pharmacologically active plant derivative;^3^: Pharmacologically active synthetic molecule; Ref: Reference

#### The case of Digoxin

Digoxin is a cardiotonic glycoside which is mainly used for the treatment of multiple heart failure related ailments^35, 36^. However, a differential therapeutic effect of this drug has been observed in different populations^16^. The structure of digoxin consists of three sugar moieties and one aglycone digoxigenin (steroid) moiety. Based on the molecular structure of digoxin; the three potential sites for metabolic reactions are C-17 attached lactone ring, 3β-OH group, and the sugar moieties. Redox reactions are known to occur at the lactone ring and 3β-OH group, whereas removal and subsequent addition are known for the sugar moieties^37^. Thus, Digoxin can be potentially metabolized by three distinct kinds of enzyme classes which are oxidoreductase (EC1) for the reduction of lactone ring, transferase (EC2) for the addition of sugar moieties and hydrolases (EC3) for the removal of sugar moieties.

The metabolism of digoxin by gut microbiota was first known almost 40 years ago where the saturation of lactone ring was reported by *ex*-*vivo* incubation with rat and human fecal samples^38^. In 1983, Saha *et al*. identified the gut bacterium ‘*Eggerthella lenta*’ (previously known as *Eubacterium lentum*) capable of reducing active digoxin into inactive dihydrodigoxin and also suggested the potential metabolism by other gut bacterial species^39, 40^. Recently, Haiser *et al*. confirmed the role of ‘*Eggerthella lenta*’ in the reduction of digoxin and also identified the operon and corresponding genes, which get over-expressed in response to a low concentration of arginine and high concentration of digoxin in two different studies^14, 40^. They reported two potential enzymes, cytochrome c reductase (Cgr1) and FAD-binding fumarate reductase (Cgr2), based on the structural and sequence homology analysis for the reduction of digoxin. In addition, the metabolism of digoxin by cytochrome P4503A (EC 1.14.14.1) was also shown^41^. Moreover, human liver alcohol dehydrogenase (EC 1.1.1.1) is also known to catalyse the oxidation of 3β-OH group of digoxin to 3-keto derivatives^42^. The cleavage of sugar moieties of digoxin due to low intragastric pH and the following conjugation reaction by hepatic UDP-glucuronyl transferase (UDPGT) (EC 2.4.1.17 and EC 2.4.1.95), which are majorly responsible for the inter-individual variability in digoxin biotransformation^43^, was also shown.

**Figure 4:**
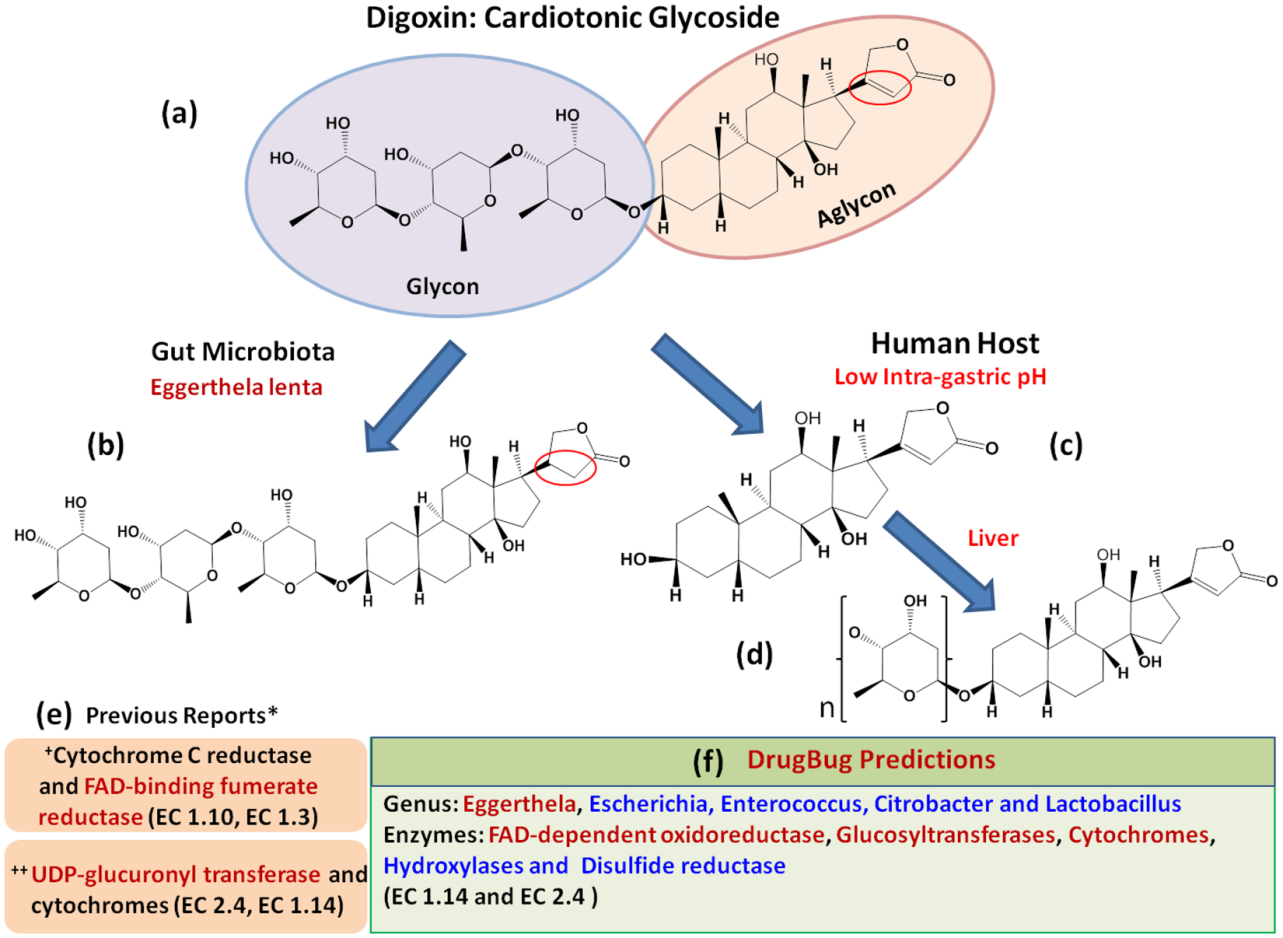
Schematic representation of digoxin metabolism. (**a**) Structure of digoxin, (**b**) Metabolism of digoxin by gut microbe, (**c**) Metabolism of digoxin at low gastric PH in human host, (**d**) Metabolism of digoxin in liver, (**e**) Previous reports on the metabolism of digoxin and (**f**) Prediction of digoxin metabolism by DrugBug approach.

Thus, taken together digoxin appears to be an interesting and important case study. To predict the potential metabolism of digoxin by gut bacteria, in the first step all the three (hybrid, fingerprinter, and pubchem) best-performing fingerprints available on the DrugBug web server were used. Both hybrid and fingerprinter predicted EC2, whereas pubchem predicted EC1 as the probable EC classes. In the second step, the output of all fingerprints was analyzed using the best-performing hybrid fingerprint which predicted EC 2.4 and EC 1.14 as the EC sub-classes. Using molecular similarity search in the third step, the predicted EC were EC2.4.1.78, EC2.4.1.- and EC1.14.13.-. The corresponding enzymes for the predicted EC2.4.1.78 and EC2.4.1.- were glucosyltransferases and mannosyltransferase from the bacterial genus Escherichia, Lactobacillus, Klebsiella, Enterococcus, and Citrobacter, whereas, for EC1.14.13.-, the corresponding enzymes were FAD-dependent oxidoreductases, disulfide reductase and hydroxylases from the bacterial genus Escherichia, Klebsiella, Providencia, Streptomyces and Eggerthella (Fig. 4).

Thus, using DrugBug, we could predict the metabolic enzymes which were already known for the metabolism of digoxin such as FAD-dependent oxidoreductases and glucosyltransferases. In addition, we could also correctly predict the EC class and sub-class (EC 2.4 and EC 1.14) known to metabolize digoxin^41, 43^ along with Eggerthella as one of the genera capable of metabolizing digoxin. However, the species predicted by DrugBug was *Eggerthela sp*. *1 3 56FAA* instead of *Eggerthela lenta* which is known to metabolize digoxin^40^. Since at the time of our local in-house database construction, the genome of *Eggerthela lenta* was not available thus, it was not included in the in-house constructed database and could not be predicted. However, the protein sequence of predicted FAD-dependent oxidoreductase of *Eggerthela sp*. *1 3 56FAA* was found to be 100% identical to the corresponding protein of *Eggerthela lenta* which confirms the accuracy of results.

## Discussion

Several experimental studies have shown that the metabolic activities of human gut bacteria are crucial for metabolism of xenobiotic/drug molecules in the human gut^14, 15^. The capability of gut bacteria to alter the pharmacokinetic and pharmacodynamic properties of orally administered drugs is especially significant in the field of pharmaceutical research, since most of the orally administered drugs are first encountered by gut microbes, which can modify the overall activity and toxicity of a drug in the human gastrointestinal tract^8–10, 44–47^. Furthermore, several metagenomic projects have recently shown that significant diversity exists in the microbial distribution and composition of gut microbiota in different populations. Thus, the knowledge of bacterial species-specific metabolism of xenobiotic/drug molecule would be helpful in predicting the possible individual-specific drug response based on the gut metagenomic profile of an individual. Identifying the potential role of gut microbiota in xenometabolism of drugs is crucial for designing more effective drug molecules; however, limited knowledge is available until today. In this scenario, the current work presents an efficient and reliable computational methodology to predict biotransformation of xenobiotic/drug molecules from the diverse and vast metabolic potential of the gut bacteria. In this work, we are predicting the enzyme from human gut bacteria which could carry out the first/initial step in the biotransformation of the given input molecule, and we are not predicting the later enzymes or the resultant metabolic products.

Furthermore, during the training set construction, the substrates which could be metabolized by enzymes from multiple EC classes were not included in the training dataset. This ensures that the training dataset contains only those substrate molecules which can be metabolized by enzymes belonging to only one out of the six EC classes. Thus, in the case of a given query drug/xenobiotic molecule which shows its best match with a substrate that is acted on by enzymes from multiple EC classes, the classifier will predict only a single EC class and sub-class for the given query molecule after the first and second steps of prediction. In the predicted EC subclass, multiple enzymes of the same subclass can be predicted through Open Babel structural similarity search. Therefore, if a molecule is a substrate for multiple enzymes, and those enzymes belong to the same EC sub-class, then DrugBug approach will predict all the enzymes. However, it will not predict the enzymes from a different EC class or EC sub-class. Nonetheless, the predictions will still be made for a query molecule similar to a substrate which was not included during training since the substrate could be metabolized by enzymes from multiple EC classes. The resultant predictions will be from a single EC class, which showed the best hit for the biotransformation of that molecule.

For the successful implementation of this approach, the construction of a comprehensive dataset of metabolic enzymes and their substrates for known gut bacteria was the key. Further, the study demonstrated that fingerprints derived from the substrate molecules could be successfully used for the development of RF models and among these, the hybrid fingerprint showed the best results. The higher performance of the with-upsampling RF models as compared to the without-upsampling RF models showed that the upsampling strategy could resolve the data imbalance issue in the original dataset, though it could also lead to some selection bias and overfitting.

We successfully developed a three-step methodology for the prediction of specific enzymes and the corresponding gut bacterial species capable of biotransforming the xenobiotic/drug molecule. To help the user to predict the metabolic enzymes and gut bacteria, the above approach has been implemented as ‘DrugBug’ web server tool where the input is the mol/sdf file of the query molecule. The current version of the DrugBug approach incorporates data from 491 human gut bacterial genomes and their 324,697 metabolic enzymes. The availability of a larger number of human gut microbial genomic sequences and their corresponding metabolic enzymes in the future is likely to improve further the accuracy, sensitivity and the scope of predictions using the DrugBug approach.

The prediction of specific enzyme and bacterial species which could potentially carry out biotransformation of the selected cases of FDA-approved drugs and other clinically important molecules further attests the significance of this approach, and provides leads for experimental validation. Thus, by using this approach, the identification of gut bacterial species and the potential enzyme which could carry out the biotransformation of a drug can be correlated with the abundance of that protein in the gut metagenome of an individual. This drug-bacteria linked metabolism would be helpful in predicting the individual-specific metabolism of that drug which is a step closer towards the goal of precision medicines. The web server is available at http://metagenomics.iiserb.ac.in/drugbug.

## Methods

To develop a tool for the prediction of gut microbial enzymes which could potentially biotransform a xenobiotic/drug molecule, two key information were required: i) a set of known microbial metabolic enzymes along with their EC numbers and ii) their corresponding substrate molecules. The above information was used for the construction of predictive machine learning random forest (RF) modules (Fig. 3a). These steps are described in detail in the following sections.

### Construction of gut bacterial enzyme database

A total of 491 available gut bacterial genomes sequences were retrieved from different sources including NCBI genomes (http://www.ncbi.nlm.nih.gov/genome/), HMP reference genomes (http://hmpdacc.org/reference_genomes/reference_genomes.php) and EMBL-EBI bacterial genomes (http://www.ebi.ac.uk/genomes/) (Supplementary Table S4). All potential metabolic enzymes from each gut bacterial genome were identified and assigned with their corresponding four-digit EC using the following strategy. The reviewed enzymatic protein sequences with their corresponding EC were downloaded from UniProt database (http://www.uniprot.org/uniprot/), and a reference database containing the EC along with their corresponding protein sequences was constructed. This database was used as the reference database for carrying out the BLAST-based protein alignment of all the proteins retrieved from different gut bacterial genomes^48^. The best hit for a gut bacterial protein was identified using the cut-off values of Identity > 40%, Query coverage > 80% and E-value < 10^−15^. The best hit could be identified for 324,697 (12.39%) proteins out of 1,571,442 total proteins, and the resultant protein sequences of metabolic enzymes were assigned with a four-digit EC as per the EC of their corresponding best hit. The identified metabolic enzymes along with their EC and bacterial genome annotation were pooled together to create a database of metabolic enzymes for the gut bacterial metagenome. Further, the taxonomy information for each bacterial genome was added to the above database.

### Construction of gut bacterial substrate database

For each bacterial metabolic enzyme, the metabolic reactions (using their EC) were fetched from the KEGG database (www.genome.jp/kegg). The substrates for the above-identified reactions were pooled together and tagged with their corresponding EC number. To prepare the substrate database, cofactors and other supporting molecules for enzyme functioning such as water, metal ions, ATP, etc. were removed by manual curation and only the principal substrate compounds were considered. This resulted in a total of 2,324 molecules in the substrate database. Further, the substrate database was divided into subsets based on their respective EC tags. These subsets were termed as “EC class-specific databases and EC subclass-specific databases.” An all-against-all structural similarity search was performed for all 2,324 molecules using Open Babel (v2.3.2) to remove redundancy and pick the representative molecules (tanimoto coefficient >0.95) for training^49^. This step was necessary to create a non-overlapping training set which is essential for the development of random forest classification models. Furthermore, the substrates which could be metabolized by enzymes belonging to multiple EC classes were also removed. Thus, out of 2,324 molecules, 1,609 representative molecules were considered for developing the RF models. The resultant 1,609 molecules were further used for the development of RF models.

### Calculation of fingerprints

For the development of RF models, the molecular information is required to be translated into machine-readable features (fixed length pattern: mostly numerical data) for each substrate molecule. To achieve the same for each molecule, ten different fingerprints were calculated using ‘PaDEL’ software^50^. The fingerprints along with their respective bit size were: Fingerprinter – 1024 bits, Estate Fingerprinter (EstateFP) – 79 bits, Graph Only Fingerprinter (GraphFP) – 1024 bits, MACCS Fingerprinter (MACCSFP) – 166 bits, PubChem Fingerprinter (PubChemFP) – 881 bits, Substructure Fingerprinter (SubFP) − 307 bits, Substructure Fingerprint Count (SubFPC) – 307 bits, Klekota Roth Fingerprinter (KRFP) – 4860 bits, Atom Pairs 2D Fingerprinter (AP2D) – 780 bits and Atom Pairs 2D Fingerprint Count (APC2D) − 780 bits. For all the 10 fingerprints, the variable importance was calculated for each bit using two attribute selection modules of Weka, i.e. Remove Useless* (re-useless) and CfsSubsetEval* with best-fit algorithm^34^. The RemoveUseless filter implemented in Weka removes the attributes (bits) that do not provide significant information such as the attributes, which do not vary or show insignificant variation. The CFS attribute subset evaluator (CfsSubsetEval) is a function implemented in Weka which carries out correlation-based subset selection of the features. This function helps to calculate the subsets of features that are highly discriminatory among the given groups.

The Weka output provided the list of bits out of the total bits present in a fingerprint that were important for classification and these bits were selected further. The selected bits of each fingerprint were combined to create a hybrid fingerprint containing a total of 219 bits (Supplementary Table S1 and Figure S5). In the subsequent analysis, the ten fingerprints and the hybrid fingerprint were considered.

### Principal Component Analysis (PCA)

Principal component analysis (PCA) is used to analyze high-dimensional data by reducing data dimensions into a manageable space, and hence, it is a powerful approach to select components in a dataset which are used to assess the variation. To compute variance among the six EC classes and the subclasses of each EC class, PCA was performed on all 1,609 molecules of substrate database using the hybrid fingerprint. The principal components were calculated using the ‘prcomp’ function in R version 3.1.2. Further, the graphs were generated using the library ‘ggbiplot’. PCA analysis was also used to find out the distribution of these molecules among different EC classes and subclasses.

### Construction of training dataset

The original substrate dataset was highly imbalanced in which 65.75% of the substrate molecules were known to be metabolized by enzymes from EC1 and EC2 classes and only 6.83% substrate molecules could be metabolized by enzymes from EC5 and EC6 classes. As it is a well-known fact that imbalanced data has a significant negative impact on the performance of RF models, a balanced dataset was created by employing upsampling strategy using ‘Caret’ package in R^51^. Upsampling method is one of the sub-sampling methods where the minority classes are up-sampled by random sampling with replacement (Text S1). Overall, two training sets were prepared- (i) original imbalanced data referred to as ‘without-upsampling data’ and (ii) the up-sampled data referred to as ‘with-upsampling data’.

### Selection and implementation of machine learning method

The performances of different machine learning classification approaches including Naïve Bayes, AdaBoost, Bagging, IBk, Multiclass classifier, Random Forest and Support Vector Machines were compared using Weka, and the results are provided in Table S2. As apparent, the best classification accuracy was shown by the random forest model.

Thus, the random forest (RF) was implemented in the study using the randomForest package in R (http://cran.r-project.org//). RF classification models are widely used methods for binary and multiclass classification of large data^52–55^. RF provides the flexibility to optimize the number of randomly selected subsets of variables (mtry) at each node, and the number of independent models (trees) in the forest^56^. At each split node, these specified subsets of variables play an important role in the calculation of variable information gain. Both the parameters, mtry and ntree, have a significant impact on the performance of the model. Thus, the mtry optimization was carried out using the tuneRF function present in the random forest package of R at a fixed ntree values of 100. The performances of RF models at optimized mtry values were further assessed at different ntree values from 100 to 500 with a step size of 100. These optimized values of mtry and ntree were used for the construction of RF models. The RF classification model constructed using optimized parameters with lowest %OOB error (error rate on out of bag data) i.e. highest prediction accuracy, was used for performance evaluation on different test datasets such as blind set and an independent set. The performance was evaluated using the following parameters.

1 $$TPR=\frac{TP}{TP+FN}$$

2 $$TNR=\frac{TN}{TN+FP}$$

3 $$PPV=\frac{TP}{TP+FP}$$

4 $$ACC=\frac{(TP+TN)}{(TP+FP+FN+TN)}$$

5 $$MCC=\frac{TP\times TN-FP\times FN}{\sqrt{(TP+FP)(TP+FN)(TN+FP)(TN+FN)}}$$

where, TP = True Positive, FP = False Positive, FN = False Negative, TN = True Negative, TPR = True Positive Rate or Sensitivity, TNR = True Negative Rate or Specificity, PPV = Positive Predictive Value or Precision, ACC = Accuracy, MCC = Matthews correlation coefficient.

### Selection of fingerprint

To select the best fingerprint for constructing the RF model, for each fingerprint the mtry values (number of randomly selected variables) at each node were optimized, and the %OOB (Out of Bag) error values were calculated at the optimized mtry values and at the ntree (number of tress in the forest) value of 500. This analysis of each fingerprint was carried out using both without-upsampling and with–upsampling training datasets. Based on the %OOB values, the best fingerprint was chosen for further RF model construction and validation.

### Construction and evaluation of RF models

Seven different RF models (one for the classification among EC classes and six for the classification among EC subclasses of each EC class) were constructed for each dataset (without-upsampling dataset and with-upsampling dataset). The RF model constructed for classification into the six EC classes was called “EC class-specific RF model”. Similarly, for each EC class, RF models were constructed for classification into the EC subclasses of that particular EC class and were termed as “EC subclass-specific RF models”. Thus, one EC class-specific and six EC subclass-specific RF models were prepared. Each RF model was evaluated using the following three evaluation methods.

#### Cross-validation

The most commonly used technique to assess the performance of a given RF model is leave-one-out cross-validation. In this study, ten-fold cross-validation strategy was used to evaluate and construct the final models. This strategy randomly divides the data into 10 equal-sized subsamples out of which nine sets are used for training, and the remaining tenth set is used for testing. This validation was performed using the function cv.fold = 10 of randomForest package in R. The overall mean performances obtained using this function for all the EC class-specific and sub-class specific RF models were reported in this study.

#### Randomly selected data for training and testing

The complete data was divided into two parts such that 75% of the data was used for training and the remaining 25% was used for testing. The performance on 25% of the data using training model was computed.

#### Blind set

The unbiased performance of the RF models was assessed using a Blind set of 162 molecules (randomly selected 10% of molecules from each EC class). These 162 molecules were removed from the training dataset and labelled as ‘Blind set’ (Supplementary Table S5). The remaining 1,447 molecules were used for the development of RF models. The performance of each RF model was evaluated on the blind set, following which these molecules were again included in the whole data, and the complete final RF models were constructed using all data (1,609 molecules).

### Molecular similarity search for the assignment of complete EC

Open Babel, a chemoinformatics tool, was used to carry out molecular similarity search for query molecules against each EC subclass-specific substrate databases. The molecular similarity was quantified using the value of similarity coefficient known as ‘tanimoto coefficient’ (formula is mentioned below). Open Babel provides three different fingerprints namely FP2, FP4 and MACCSFP for calculating the respective tanimoto coefficient. If two or more than two fingerprints out of the three gave the same molecule as the top hit, then that molecule was considered as the molecule similar to the query. In cases where the three fingerprints provided three different molecules as the top hit, the molecule with the highest tanimoto coefficient was considered as most similar to the query molecule. Tanimoto coefficient between any two molecules (e.g. X and Y) can be calculated using the following formula.

6 $$TC=\frac{z}{x+y-z}$$

where, TC = Tanimoto coefficient, x = number of bits set to 1 in X, y = number of bits set to 1 in Y and z = number of bits set to 1 in both X and Y^57^.

### Prediction of gut bacteria and metabolic enzyme

Using the above step, the four-digit EC number (enzyme) is identified, which can carry out the biotransformation of a query molecule if the query molecule shows structural similarity with its known substrate. Considering the promiscuous nature of the metabolic enzymes, all the gut microbial metabolic enzymes belonging to a particular four-digit EC number were identified as the metabolic enzymes capable of biotransforming the xenobiotic/drug molecule. Furthermore, all the gut bacteria harbouring the identified enzymes were considered as the gut bacteria capable of metabolizing the query molecule.

## Electronic supplementary material

Supplementary Information
