# Supplementary material for: A novel approach for the prediction of species-specific biotransformation of xenobiotic/drug molecules by the human gut microbiota
Source: Sci Rep. 2017 Aug 29;7:9751. doi: 10.1038/s41598-017-10203-6 (PMC5575299; doi:10.1038/s41598-017-10203-6)

**Title: A novel approach for the prediction of species-specific biotransformation of xenobiotic/drug molecules by human gut microbiota**

**Authors:** Ashok K. Sharma*, Shubham K. Jaiswal*, Nikhil Chaudhary, Vineet K. Sharma

**S1 Text. Description of Upsampling strategy**

Up-sampling in the datasets was implemented using upSample function of caret package in R. It is described in detail in the following text.

<https://rdrr.io/cran/caret/man/downSample.html>

[*http://finzi.psych.upenn.edu/R/library/caret/html/downSample.html*](http://finzi.psych.upenn.edu/R/library/caret/html/downSample.html)

Random sampling with replacement, which is carried out during upsampling, is a known statistical re-sampling method where the term ‘random sampling’ implies that each individual data point of the dataset has an equal chance of being selected, and ‘sampling with replacement’ implies that each data point of the dataset can be selected more than once. Therefore, random sampling with replacement collectively implies that during re-sampling each data point has equal probability to get selected and each data point can be selected multiple numbers of times. The number of instances are increased by selecting the same data points multiple number of times, and this is done randomly to remove any biasness due to the selection of a particular data point multiple of times.

In this work, upsampling of the minority classes was carried out to make the number of instances in those classes equal to the highest represented class to resolve the class imbalance issue.

Up-sampling/Over-sampling is a well-known strategy to remove data imbalance problem. Several previous studies have utilized it for different kinds of datasets, such as a) cases versus control data in the autism prediction, b) on cardiovascular medical data to predict the risk of cardiovascular failure or related disease, c) behavioural data for the acoustic emotion recognition, d) pixels data for the image restoration with convolutional networks. The references for these studies are mentioned in the following text. (<http://www.nature.com/tp/journal/v2/n4/full/tp201210a.html>, <http://www.ijmlc.org/papers/307-K0020.pdf>, <http://citeseerx.ist.psu.edu/viewdoc/download?doi=10.1.1.364.4889&rep=rep1&type=pdf>, <http://ieeexplore.ieee.org/xpl/login.jsp?tp=&arnumber=4408909&url=http%3A%2F%2Fieeexplore.ieee.org%2Fxpls%2Fabs_all.jsp%3Farnumber%3D4408909>).

*Performance of undersampling methodology*

The performance of RF model on the dataset using random undersampling of the majority classes using the ‘downSample’ function of the caret package in R was also examined. At the optimized parameters, the CV-10 fold classification performance obtained using under-sampled data was lower (MCC = 0.39) in comparison to the original data (MCC =0.49, Table 1), and was much lower in comparison to the up-sampled data (MCC =0.89, Table 1). Thus, the undersampling was not considered in the subsequent analysis.

**S2 Text. Details of modules construction**

After obtaining a set of substrate molecules for all gut microbial metabolic enzymes, the first step is the construction of prediction models to predict the EC class and subclass capable of carrying out the biotransformation of a query molecule. Machine learning implemented using Random Forest (RF) was used to construct the prediction module and similarity searching module was implemented using Open Babel. The two modules were used serially where the output of machine learning based module was used as the input for similarity searching module. The inputs and outputs of RF modules are described below.

***RF module 1***

This module was trained on fingerprints derived from all substrate molecules present in the substrate database of all six EC classes. The details of fingerprints calculation are provided in Figure S5 and manuscript text.

This module predicts the EC class capable of carrying out the biotransformation of a query molecule.

***RF module 2***

RF modules were constructed for each of the EC classes, and were trained on fingerprints of substrate molecules belonging to specific EC subclass.

This module predicts the EC subclass capable of carrying out the biotransformation of a query molecule.

The output of RF modules is EC class and subclass from which the enzymes are capable of carrying out the biotransformation of a drug molecule. Finally, the four-digit EC number of the metabolizing enzymes is found using similarity search by Open Babel which compares the structure of a given query molecule with all substrate structures known for the enzymes of the predicted EC subclass.

**S3 Text. Selection of threshold values for the prediction of EC class and EC subclass**

Prediction of EC class and EC subclass is carried out in the first two steps of DrugBug approach based on a probability score provided by random forest. The web server provides the user with an option to choose a probability threshold value, else the default value of 0.5 is used. If the predictions are below the threshold, the program will pause with a warning message that the prediction probability is below the selected threshold value. However, at this step the user is also provided with an option to proceed further with the analysis.

At the similarity search step also, the user has the option to select a particular tanimoto coefficient threshold value so that only the results above this value are displayed at the result page. Thus, the user has options to select the custom threshold values at all three steps of DrugBug which allows the user to decide upon the likelihood of the metabolism of the drug through the different resultant enzymes. In general, higher the prediction probability and the tanimoto coefficient value, the higher will be the likelihood of the resultant enzyme to act on the input drug molecule.

However, for cases where the prediction probability values at the first two steps which determine the EC class and subclass, and the tanimoto coefficient value at the similarity search step are too low, it is likely that the input drug molecule may not be metabolized through the gut bacterial enzymes.

**Table S1.** Number of selected attributes using Weka from each fingerprint for the construction of hybrid set.

| **Fingerprints** | **Important attributes** |
| --- | --- |
| **AD2D** | 8 |
| **APC2D** | 15 |
| **Estate** | 8 |
| **Finger Printer** | 51 |
| **GraphFP** | 27 |
| **KRFP** | 34 |
| **MACCSFP** | 23 |
| **Pubchem** | 24 |
| **SubFP** | 13 |
| **SubFPC** | 16 |

**Table S2.** Performance comparison of different machine learning methods using Weka.

| **Methods** | **Total instances** | **Correctly classified instances (%)** |
| --- | --- | --- |
| **Naïve Bayes** | 1609 | 52.14 |
| **AdaBoost** | 1609 | 48.85 |
| **Bagging** | 1609 | 63.57 |
| **IBk** | 1609 | 63.57 |
| **MultiClass classifier** | 1609 | 54.44 |
| **Random Forest** | 1609 | 64.57 |
| **SVM** | 1609 | 55.06 |

**Table S3.** Optimization of ntree value for the construction of RF models.

| **Ntree** | **% OOB** |
| --- | --- |
| **100** | 8.52 |
| **200** | 8.61 |
| **300** | 8.55 |
| **400** | 8.44 |
| **500** | 8.42 |

**Table S4.** List of 491 gut bacterial genomes which were used for the construction of metabolic enzyme and substrate databases.

| **Bacterial Genome** | **Source** |
| --- | --- |
| **Bacteroides_dorei_5_1_36/D4** | HMP Gastrointestinal Tract |
| **Bacteroides_fragilis_NCTC_9343** | NCBI/PMC4822603/ |
| **Bacteroides_fragilis_YCH46** | NCBI/doi:10.1371/journal.pone.005382 |
| **Bacteroides_vulgatus_ATCC_8482** | NCBI/PMID: 27040269 |
| **Bifidobacterium_adolescentis_ATCC_15703** | NCBI/JGI-GOLD(Gp0000246) |
| **Bifidobacterium_animalis_subsp._lactis_AD011** | EMBL-EBI/doi:10.1128/JB.01515-08 |
| **Bifidobacterium_longum_DJO10A** | NCBI/UniProt(UP000002419) |
| **Bifidobacterium_longum_NCC2705** | NCBI/PMID: 19534605 |
| **Butyrivibrio_fibrisolvens_16/4** | HMP Gastrointestinal Tract |
| **Clostridiales_butyrate-producing_bacterium_SM4/1** | HMP Gastrointestinal Tract |
| **Clostridiales_butyrate-producing_bacterium_SS3/4** | HMP Gastrointestinal Tract |
| **Clostridiales_butyrate-producing_bacterium_SSC/2** | HMP Gastrointestinal Tract |
| **Clostridium_sp._M62/1** | HMP Gastrointestinal Tract |
| **Clostridium_sp._SS2/1** | HMP Gastrointestinal Tract |
| **Coprococcus_catus_GD/7** | HMP Gastrointestinal Tract |
| **Coprococcus_sp._ART55/1** | HMP Gastrointestinal Tract |
| **Enterococcus_casseliflavus_EC20** | NCBI/tRNADB-CE(ACAO01000051) |
| **Escherichia_coli_536** | NCBI/doi:10.1371/journal.pone.00060 |
| **Escherichia_coli_APEC_O1** | NCBI/doi:10.1371/journal.pone.00060 |
| **Escherichia_coli_ATCC_8739** | NCBI/doi:10.1371/journal.pone.00060 |
| **Escherichia_coli_CFT073** | NCBI/doi:10.1371/journal.pone.00060 |
| **Escherichia_coli_ED1a** | NCBI/doi:10.1371/journal.pone.00060 |
| **Escherichia_coli_HS** | NCBI/doi:10.1371/journal.pone.00060 |
| **Escherichia_coli_IAI39** | NCBI/doi:10.1371/journal.pone.00060 |
| **Escherichia_coli_O127:H6_str._E2348/69** | EMBL-EBI/doi:10.1371/journal.pone.00060 |
| **Escherichia_coli_S88** | NCBI/doi:10.1371/journal.pone.00060 |
| **Escherichia_coli_UTI89** | NCBI/doi:10.1371/journal.pone.00060 |
| **Eubacterium_rectale_M104/1** | HMP Gastrointestinal Tract |
| **Eubacterium_siraeum_70/3** | HMP Gastrointestinal Tract |
| **Faecalibacterium_prausnitzii_M21/2** | HMP Gastrointestinal Tract |
| **Faecalibacterium_prausnitzii_SL3/3** | HMP Gastrointestinal Tract |
| **Klebsiella_pneumoniae_342** | NCBI/PMID: 1865463 |
| **Klebsiella_pneumoniae_subsp._pneumoniae_MGH_78578** | EMBL-EBI/doi:10.1371/journal.pone.00060 |
| **Lactobacillus_acidophilus_NCFM** | NCBI/PMID: 23363771 |
| **Lactobacillus_brevis_ATCC_367** | NCBI/doi: 10.1371/journal.pone.0060521 |
| **Lactobacillus_casei_ATCC_334** | NCBI/PMID: 23363771 |
| **Lactobacillus_casei_BL23** | NCBI/PMID: 23363771 |
| **Lactobacillus_gasseri_ATCC_33323** | NCBI/PMID: 23363771 |
| **Lactobacillus_helveticus_DPC_4571** | NCBI/doi:10.3389/fmicb.2012.0 |
| **Lactobacillus_johnsonii_NCC_533** | NCBI/PMID: 23363771 |
| **Lactobacillus_plantarum_WCFS1** | NCBI/doi:10.1128/JB.06275-11 |
| **Lactobacillus_reuteri_DSM_20016** | NCBI/PMID: 23363771 |
| **Lactobacillus_sakei_subsp._sakei_23K** | EMBL-EBI/UniProt(UP000002707) |
| **Lactobacillus_salivarius_UCC118** | NCBI/doi:10.1371/journal.pone.00060 |
| **Megamonas_hypermegale_ART12/1** | HMP Gastrointestinal Tract |
| **Roseburia_intestinalis_M50/1** | HMP Gastrointestinal Tract |
| **Ruminococcus_sp._SR1/5** | HMP Gastrointestinal Tract |
| **Acidaminococcus_sp._D21** | HMP Gastrointestinal Tract |
| **Acidaminococcus_sp._HPA0509** | HMP Gastrointestinal Tract |
| **Acinetobacter_junii_SH205** | HMP Gastrointestinal Tract |
| **Acinetobacter_radioresistens_SH164** | HMP Gastrointestinal Tract |
| **Actinomyces_odontolyticus_ATCC_17982** | NCBI/JGI-GOLD(Gp0000877) |
| **Actinomyces_sp._HPA0247** | HMP Gastrointestinal Tract |
| **Akkermansia_muciniphila_ATCC_BAA-835** | EMBL-EBI/DOI10.1099/ijs.0.02873-0 |
| **Alistipes_indistinctus_YIT_12060** | HMP Gastrointestinal Tract |
| **Alistipes_putredinis_DSM_17216** | HMP Gastrointestinal Tract |
| **Alistipes_shahii_WAL_8301** | HMP Gastrointestinal Tract |
| **Anaerobaculum_hydrogeniformans_ATCC_BAA-1850** | HMP Gastrointestinal Tract |
| **Anaerococcus_hydrogenalis_DSM_7454** | HMP Gastrointestinal Tract |
| **Anaerofustis_stercorihominis_DSM_17244** | HMP Gastrointestinal Tract |
| **Anaerostipes_caccae_DSM_14662** | HMP Gastrointestinal Tract |
| **Anaerostipes_hadrus_DSM_3319** | HMP Gastrointestinal Tract |
| **Anaerostipes_sp._3_2_56FAA** | HMP Gastrointestinal Tract |
| **Anaerotruncus_colihominis_DSM_17241** | HMP Gastrointestinal Tract |
| **Arcobacter_butzleri_JV22** | HMP Gastrointestinal Tract |
| **Bacillus_smithii_7_3_47FAA** | HMP Gastrointestinal Tract |
| **Bacillus_sp._7_6_55CFAA_CT2** | HMP Gastrointestinal Tract |
| **Bacteroides_caccae_ATCC_43185** | HMP Gastrointestinal Tract |
| **Bacteroides_cellulosilyticus_DSM_14838** | HMP Gastrointestinal Tract |
| **Bacteroides_clarus_YIT_12056** | HMP Gastrointestinal Tract |
| **Bacteroides_coprocola_DSM_17136** | HMP Gastrointestinal Tract |
| **Bacteroides_coprophilus_DSM_18228_=_JCM_13818** | HMP Gastrointestinal Tract |
| **Bacteroides_dorei_DSM_17855** | HMP Gastrointestinal Tract |
| **Bacteroides_eggerthii_1_2_48FAA** | HMP Gastrointestinal Tract |
| **Bacteroides_eggerthii_DSM_20697** | HMP Gastrointestinal Tract |
| **Bacteroides_finegoldii_DSM_17565** | HMP Gastrointestinal Tract |
| **Bacteroides_fluxus_YIT_12057** | HMP Gastrointestinal Tract |
| **Bacteroides_fragilis_3_1_12** | HMP Gastrointestinal Tract |
| **Bacteroides_intestinalis_DSM_17393** | HMP Gastrointestinal Tract |
| **Bacteroides_oleiciplenus_YIT_12058** | HMP Gastrointestinal Tract |
| **Bacteroides_ovatus_3_8_47FAA** | HMP Gastrointestinal Tract |
| **Bacteroides_ovatus_ATCC_8483** | HMP Gastrointestinal Tract |
| **Bacteroides_ovatus_SD_CMC_3f** | HMP Gastrointestinal Tract |
| **Bacteroides_pectinophilus_ATCC_43243** | HMP Gastrointestinal Tract |
| **Bacteroides_plebeius_DSM_17135** | HMP Gastrointestinal Tract |
| **Bacteroides_salyersiae_WAL_10018_=_DSM_18765_=_JCM_12988** | HMP Gastrointestinal Tract |
| **Bacteroides_sp._1_1_14** | HMP Gastrointestinal Tract |
| **Bacteroides_sp._1_1_30** | HMP Gastrointestinal Tract |
| **Bacteroides_sp._1_1_6** | HMP Gastrointestinal Tract |
| **Bacteroides_sp._2_1_16** | HMP Gastrointestinal Tract |
| **Bacteroides_sp._2_1_22** | HMP Gastrointestinal Tract |
| **Bacteroides_sp._2_1_33B** | HMP Gastrointestinal Tract |
| **Bacteroides_sp._2_1_56FAA** | HMP Gastrointestinal Tract |
| **Bacteroides_sp._2_2_4** | HMP Gastrointestinal Tract |
| **Bacteroides_sp._3_1_19** | HMP Gastrointestinal Tract |
| **Bacteroides_sp._3_1_23** | HMP Gastrointestinal Tract |
| **Bacteroides_sp._3_1_33FAA** | HMP Gastrointestinal Tract |
| **Bacteroides_sp._3_1_40A** | HMP Gastrointestinal Tract |
| **Bacteroides_sp._3_2_5** | HMP Gastrointestinal Tract |
| **Bacteroides_sp._4_1_36** | HMP Gastrointestinal Tract |
| **Bacteroides_sp._4_3_47FAA** | HMP Gastrointestinal Tract |
| **Bacteroides_sp._9_1_42FAA** | HMP Gastrointestinal Tract |
| **Bacteroides_sp._D1** | HMP Gastrointestinal Tract |
| **Bacteroides_sp._D20** | HMP Gastrointestinal Tract |
| **Bacteroides_sp._D22** | HMP Gastrointestinal Tract |
| **Bacteroides_sp._HPS0048** | HMP Gastrointestinal Tract |
| **Bacteroides_stercoris_ATCC_43183** | HMP Gastrointestinal Tract |
| **Bacteroides_thetaiotaomicron_VPI-5482** | EMBL-EBI/PMID: 12663928 |
| **Bacteroides_uniformis_ATCC_8492** | HMP Gastrointestinal Tract |
| **Bacteroides_vulgatus_PC510** | HMP Gastrointestinal Tract |
| **Bacteroides_xylanisolvens_SD_CC_1b** | HMP Gastrointestinal Tract |
| **Bacteroides_xylanisolvens_SD_CC_2a** | HMP Gastrointestinal Tract |
| **Bacteroides_xylanisolvens_XB1A** | HMP Gastrointestinal Tract |
| **Barnesiella_intestinihominis_YIT_11860** | HMP Gastrointestinal Tract |
| **Bifidobacterium_adolescentis_L2-32** | HMP Gastrointestinal Tract |
| **Bifidobacterium_angulatum_DSM_20098_=_JCM_7096** | HMP Gastrointestinal Tract |
| **Bifidobacterium_bifidum_NCIMB_41171** | HMP Gastrointestinal Tract |
| **Bifidobacterium_breve_DSM_20213_=_JCM_1192** | HMP Gastrointestinal Tract |
| **Bifidobacterium_breve_HPH0326** | HMP Gastrointestinal Tract |
| **Bifidobacterium_catenulatum_DSM_16992_=_JCM_1194_=_LMG_11043** | HMP Gastrointestinal Tract |
| **Bifidobacterium_dentium_ATCC_27678** | HMP Gastrointestinal Tract |
| **Bifidobacterium_gallicum_DSM_20093_=_LMG_11596** | HMP Gastrointestinal Tract |
| **Bifidobacterium_longum_subsp._infantis_157F** | HMP Gastrointestinal Tract |
| **Bifidobacterium_longum_subsp._infantis_ATCC_15697_=_JCM_1222_=_DSM_20088** | HMP Gastrointestinal Tract |
| **Bifidobacterium_longum_subsp._infantis_CCUG_52486** | HMP Gastrointestinal Tract |
| **Bifidobacterium_longum_subsp._longum_2-2B** | HMP Gastrointestinal Tract |
| **Bifidobacterium_longum_subsp._longum_44B** | HMP Gastrointestinal Tract |
| **Bifidobacterium_longum_subsp._longum_ATCC_55813** | HMP Gastrointestinal Tract |
| **Bifidobacterium_longum_subsp._longum_F8** | HMP Gastrointestinal Tract |
| **Bifidobacterium_longum_subsp._longum_JCM_1217** | HMP Gastrointestinal Tract |
| **Bifidobacterium_pseudocatenulatum_DSM_20438_=_JCM_1200_=_LMG_10505** | HMP Gastrointestinal Tract |
| **Bifidobacterium_sp._12_1_47BFAA** | HMP Gastrointestinal Tract |
| **Bilophila_sp._4_1_30** | HMP Gastrointestinal Tract |
| **Bilophila_wadsworthia_3_1_6** | HMP Gastrointestinal Tract |
| **Blautia_hansenii_DSM_20583** | HMP Gastrointestinal Tract |
| **Blautia_hydrogenotrophica_DSM_10507** | HMP Gastrointestinal Tract |
| **Burkholderiales_bacterium_1_1_47** | HMP Gastrointestinal Tract |
| **Butyricicoccus_pullicaecorum_1.2** | HMP Gastrointestinal Tract |
| **Butyrivibrio_crossotus_DSM_2876** | HMP Gastrointestinal Tract |
| **Campylobacter_coli_JV20** | HMP Gastrointestinal Tract |
| **Campylobacter_sp._10_1_50** | HMP Gastrointestinal Tract |
| **Campylobacter_upsaliensis_JV21** | HMP Gastrointestinal Tract |
| **Catenibacterium_mitsuokai_DSM_15897** | HMP Gastrointestinal Tract |
| **Cedecea_davisae_DSM_4568** | HMP Gastrointestinal Tract |
| **Citrobacter_freundii_4_7_47CFAA** | HMP Gastrointestinal Tract |
| **Citrobacter_sp._30_2** | HMP Gastrointestinal Tract |
| **Citrobacter_youngae_ATCC_29220** | HMP Gastrointestinal Tract |
| **Clostridiales_bacterium_1_7_47FAA** | HMP Gastrointestinal Tract |
| **Clostridium_asparagiforme_DSM_15981** | HMP Gastrointestinal Tract |
| **Clostridium_bolteae_ATCC_BAA-613** | HMP Gastrointestinal Tract |
| **Clostridium_celatum_DSM_1785** | HMP Gastrointestinal Tract |
| **Clostridium_cf._saccharolyticum_K10** | HMP Gastrointestinal Tract |
| **Clostridium_citroniae_WAL-17108** | HMP Gastrointestinal Tract |
| **Clostridium_clostridioforme_2_1_49FAA** | HMP Gastrointestinal Tract |
| **Clostridium_hathewayi_DSM_13479** | HMP Gastrointestinal Tract |
| **Clostridium_hathewayi_WAL-18680** | HMP Gastrointestinal Tract |
| **Clostridium_hiranonis_DSM_13275** | HMP Gastrointestinal Tract |
| **Clostridium_hylemonae_DSM_15053** | HMP Gastrointestinal Tract |
| **Clostridium_leptum_DSM_753** | HMP Gastrointestinal Tract |
| **Clostridium_methylpentosum_DSM_5476** | HMP Gastrointestinal Tract |
| **Clostridium_perfringens_WAL-14572** | HMP Gastrointestinal Tract |
| **Clostridium_scindens_ATCC_35704** | HMP Gastrointestinal Tract |
| **Clostridium_sp._7_2_43FAA** | HMP Gastrointestinal Tract |
| **Clostridium_sp._7_3_54FAA** | HMP Gastrointestinal Tract |
| **Clostridium_sp._D5** | HMP Gastrointestinal Tract |
| **Clostridium_sp._HGF2** | HMP Gastrointestinal Tract |
| **Clostridium_spiroforme_DSM_1552** | HMP Gastrointestinal Tract |
| **Clostridium_sp._L2-50** | HMP Gastrointestinal Tract |
| **Clostridium_sporogenes_ATCC_15579** | HMP Gastrointestinal Tract |
| **Clostridium_symbiosum_WAL-14163** | HMP Gastrointestinal Tract |
| **Clostridium_symbiosum_WAL-14673** | HMP Gastrointestinal Tract |
| **Collinsella_aerofaciens_ATCC_25986** | HMP Gastrointestinal Tract |
| **Collinsella_intestinalis_DSM_13280** | HMP Gastrointestinal Tract |
| **Collinsella_stercoris_DSM_13279** | HMP Gastrointestinal Tract |
| **Collinsella_tanakaei_YIT_12063** | HMP Gastrointestinal Tract |
| **Coprobacillus_sp._29_1** | HMP Gastrointestinal Tract |
| **Coprobacillus_sp._3_3_56FAA** | HMP Gastrointestinal Tract |
| **Coprobacillus_sp._8_2_54BFAA** | HMP Gastrointestinal Tract |
| **Coprobacillus_sp._D7** | HMP Gastrointestinal Tract |
| **Coprococcus_comes_ATCC_27758** | HMP Gastrointestinal Tract |
| **Coprococcus_eutactus_ATCC_27759** | HMP Gastrointestinal Tract |
| **Coprococcus_sp._HPP0048** | HMP Gastrointestinal Tract |
| **Coprococcus_sp._HPP0074** | HMP Gastrointestinal Tract |
| **Corynebacterium_ammoniagenes_DSM_20306** | HMP Gastrointestinal Tract |
| **Corynebacterium_sp._HFH0082** | HMP Gastrointestinal Tract |
| **Dermabacter_sp._HFH0086** | HMP Gastrointestinal Tract |
| **Desulfitobacterium_hafniense_DP7** | HMP Gastrointestinal Tract |
| **Desulfovibrio_piger_ATCC_29098** | HMP Gastrointestinal Tract |
| **Desulfovibrio_sp._3_1_syn3** | HMP Gastrointestinal Tract |
| **Desulfovibrio_sp._6_1_46AFAA** | HMP Gastrointestinal Tract |
| **Dialister_succinatiphilus_YIT_11850** | HMP Gastrointestinal Tract |
| **Dorea_formicigenerans_4_6_53AFAA** | HMP Gastrointestinal Tract |
| **Dorea_formicigenerans_ATCC_27755** | HMP Gastrointestinal Tract |
| **Dorea_longicatena_DSM_13814** | HMP Gastrointestinal Tract |
| **Dysgonomonas_gadei_ATCC_BAA-286** | HMP Gastrointestinal Tract |
| **Dysgonomonas_mossii_DSM_22836** | HMP Gastrointestinal Tract |
| **Edwardsiella_tarda_ATCC_23685** | HMP Gastrointestinal Tract |
| **Eggerthella_sp._1_3_56FAA** | HMP Gastrointestinal Tract |
| **Eggerthella_sp._HGA1** | HMP Gastrointestinal Tract |
| **Enterobacter_cancerogenus_ATCC_35316** | HMP Gastrointestinal Tract |
| **Enterobacter_cloacae_subsp._cloacae_NCTC_9394** | HMP Gastrointestinal Tract |
| **Enterobacteriaceae_bacterium_9_2_54FAA** | HMP Gastrointestinal Tract |
| **Enterococcus_faecalis_PC1.1** | HMP Gastrointestinal Tract |
| **Enterococcus_faecalis_TX0104** | HMP Gastrointestinal Tract |
| **Enterococcus_faecalis_TX1302** | HMP Gastrointestinal Tract |
| **Enterococcus_faecalis_TX1322** | HMP Gastrointestinal Tract |
| **Enterococcus_faecalis_TX1341** | HMP Gastrointestinal Tract |
| **Enterococcus_faecalis_TX1342** | HMP Gastrointestinal Tract |
| **Enterococcus_faecalis_TX1346** | HMP Gastrointestinal Tract |
| **Enterococcus_faecalis_TX1467** | HMP Gastrointestinal Tract |
| **Enterococcus_faecalis_TX2134** | HMP Gastrointestinal Tract |
| **Enterococcus_faecalis_TX2137** | HMP Gastrointestinal Tract |
| **Enterococcus_faecalis_TX4244** | HMP Gastrointestinal Tract |
| **Enterococcus_faecium_PC4.1** | HMP Gastrointestinal Tract |
| **Enterococcus_faecium_TX1330** | HMP Gastrointestinal Tract |
| **Enterococcus_saccharolyticus_30_1** | HMP Gastrointestinal Tract |
| **Enterococcus_sp._7L76** | HMP Gastrointestinal Tract |
| **Erysipelatoclostridium_ramosum_DSM_1402** | HMP Gastrointestinal Tract |
| **Erysipelotrichaceae_bacterium_21_3** | HMP Gastrointestinal Tract |
| **Erysipelotrichaceae_bacterium_2_2_44A** | HMP Gastrointestinal Tract |
| **Erysipelotrichaceae_bacterium_3_1_53** | HMP Gastrointestinal Tract |
| **Erysipelotrichaceae_bacterium_5_2_54FAA** | HMP Gastrointestinal Tract |
| **Erysipelotrichaceae_bacterium_6_1_45** | HMP Gastrointestinal Tract |
| **Escherichia_coli_4_1_47FAA** | HMP Gastrointestinal Tract |
| **Escherichia_coli_D9** | HMP Gastrointestinal Tract |
| **Escherichia_coli_MS_107-1** | HMP Gastrointestinal Tract |
| **Escherichia_coli_MS_110-3** | HMP Gastrointestinal Tract |
| **Escherichia_coli_MS_115-1** | HMP Gastrointestinal Tract |
| **Escherichia_coli_MS_116-1** | HMP Gastrointestinal Tract |
| **Escherichia_coli_MS_117-3** | HMP Gastrointestinal Tract |
| **Escherichia_coli_MS_119-7** | HMP Gastrointestinal Tract |
| **Escherichia_coli_MS_124-1** | HMP Gastrointestinal Tract |
| **Escherichia_coli_MS_145-7** | HMP Gastrointestinal Tract |
| **Escherichia_coli_MS_146-1** | HMP Gastrointestinal Tract |
| **Escherichia_coli_MS_153-1** | HMP Gastrointestinal Tract |
| **Escherichia_coli_MS_16-3** | HMP Gastrointestinal Tract |
| **Escherichia_coli_MS_175-1** | HMP Gastrointestinal Tract |
| **Escherichia_coli_MS_182-1** | HMP Gastrointestinal Tract |
| **Escherichia_coli_MS_185-1** | HMP Gastrointestinal Tract |
| **Escherichia_coli_MS_187-1** | HMP Gastrointestinal Tract |
| **Escherichia_coli_MS_196-1** | HMP Gastrointestinal Tract |
| **Escherichia_coli_MS_198-1** | HMP Gastrointestinal Tract |
| **Escherichia_coli_MS_200-1** | HMP Gastrointestinal Tract |
| **Escherichia_coli_MS_21-1** | HMP Gastrointestinal Tract |
| **Escherichia_coli_MS_45-1** | HMP Gastrointestinal Tract |
| **Escherichia_coli_MS_57-2** | HMP Gastrointestinal Tract |
| **Escherichia_coli_MS_60-1** | HMP Gastrointestinal Tract |
| **Escherichia_coli_MS_69-1** | HMP Gastrointestinal Tract |
| **Escherichia_coli_MS_78-1** | HMP Gastrointestinal Tract |
| **Escherichia_coli_MS_79-10** | HMP Gastrointestinal Tract |
| **Escherichia_coli_MS_84-1** | HMP Gastrointestinal Tract |
| **Escherichia_coli_MS_85-1** | HMP Gastrointestinal Tract |
| **Escherichia_coli_SE11** | HMP Gastrointestinal Tract |
| **Escherichia_coli_SE15** | HMP Gastrointestinal Tract |
| **Escherichia_coli_SMS-3-5** | EMBL-EBI/doi:10.1371/journal.pone.00060 |
| **Escherichia_sp._1_1_43** | HMP Gastrointestinal Tract |
| **Escherichia_sp._3_2_53FAA** | HMP Gastrointestinal Tract |
| **Escherichia_sp._4_1_40B** | HMP Gastrointestinal Tract |
| **Eubacterium_biforme_DSM_3989** | HMP Gastrointestinal Tract |
| **Eubacterium_cylindroides_T2-87** | HMP Gastrointestinal Tract |
| **Eubacterium_dolichum_DSM_3991** | HMP Gastrointestinal Tract |
| **Eubacterium_hallii_DSM_3353** | HMP Gastrointestinal Tract |
| **Eubacterium_rectale_DSM_17629** | HMP Gastrointestinal Tract |
| **Eubacterium_siraeum_DSM_15702** | HMP Gastrointestinal Tract |
| **Eubacterium_siraeum_V10Sc8a** | HMP Gastrointestinal Tract |
| **Eubacterium_sp._3_1_31** | HMP Gastrointestinal Tract |
| **Eubacterium_ventriosum_ATCC_27560** | HMP Gastrointestinal Tract |
| **Faecalibacterium_cf._prausnitzii_KLE1255** | HMP Gastrointestinal Tract |
| **Faecalibacterium_prausnitzii_A2-165** | HMP Gastrointestinal Tract |
| **Faecalibacterium_prausnitzii_L2-6** | HMP Gastrointestinal Tract |
| **Finegoldia_magna_ATCC_29328** | HMP Gastrointestinal Tract |
| **Flavonifractor_plautii_ATCC_29863** | HMP Gastrointestinal Tract |
| **Fusobacterium_gonidiaformans_3-1-5R** | HMP Gastrointestinal Tract |
| **Fusobacterium_gonidiaformans_ATCC_25563** | HMP Gastrointestinal Tract |
| **Fusobacterium_mortiferum_ATCC_9817** | HMP Gastrointestinal Tract |
| **Fusobacterium_necrophorum_D12** | HMP Gastrointestinal Tract |
| **Fusobacterium_necrophorum_subsp._funduliforme_1_1_36S** | HMP Gastrointestinal Tract |
| **Fusobacterium_nucleatum_subsp._animalis_11_3_2** | HMP Gastrointestinal Tract |
| **Fusobacterium_nucleatum_subsp._animalis_21_1A** | HMP Gastrointestinal Tract |
| **Fusobacterium_nucleatum_subsp._animalis_3_1_33** | HMP Gastrointestinal Tract |
| **Fusobacterium_nucleatum_subsp._animalis_7_1** | HMP Gastrointestinal Tract |
| **Fusobacterium_nucleatum_subsp._animalis_ATCC_51191** | HMP Gastrointestinal Tract |
| **Fusobacterium_nucleatum_subsp._animalis_D11** | HMP Gastrointestinal Tract |
| **Fusobacterium_nucleatum_subsp._vincentii_4_1_13** | HMP Gastrointestinal Tract |
| **Fusobacterium_periodonticum_1_1_41FAA** | HMP Gastrointestinal Tract |
| **Fusobacterium_periodonticum_2_1_31** | HMP Gastrointestinal Tract |
| **Fusobacterium_ulcerans_12-1B** | HMP Gastrointestinal Tract |
| **Fusobacterium_ulcerans_ATCC_49185** | HMP Gastrointestinal Tract |
| **Fusobacterium_varium_ATCC_27725** | HMP Gastrointestinal Tract |
| **Gordonibacter_pamelaeae_7-10-1-b** | HMP Gastrointestinal Tract |
| **Hafnia_alvei_ATCC_51873** | HMP Gastrointestinal Tract |
| **Helicobacter_bilis_ATCC_43879** | HMP Gastrointestinal Tract |
| **Helicobacter_canadensis_MIT_98-5491** | HMP Gastrointestinal Tract |
| **Helicobacter_cinaedi_CCUG_18818** | HMP Gastrointestinal Tract |
| **Helicobacter_pullorum_MIT_98-5489** | HMP Gastrointestinal Tract |
| **Helicobacter_pylori_35A** | HMP Gastrointestinal Tract |
| **Helicobacter_pylori_83** | HMP Gastrointestinal Tract |
| **Helicobacter_pylori_GAM100Ai** | HMP Gastrointestinal Tract |
| **Helicobacter_pylori_GAM101Biv** | HMP Gastrointestinal Tract |
| **Helicobacter_pylori_GAM103Bi** | HMP Gastrointestinal Tract |
| **Helicobacter_pylori_GAM105Ai** | HMP Gastrointestinal Tract |
| **Helicobacter_pylori_GAM112Ai** | HMP Gastrointestinal Tract |
| **Helicobacter_pylori_GAM114Ai** | HMP Gastrointestinal Tract |
| **Helicobacter_pylori_GAM115Ai** | HMP Gastrointestinal Tract |
| **Helicobacter_pylori_GAM118Bi** | HMP Gastrointestinal Tract |
| **Helicobacter_pylori_GAM119Bi** | HMP Gastrointestinal Tract |
| **Helicobacter_pylori_GAM120Ai** | HMP Gastrointestinal Tract |
| **Helicobacter_pylori_GAM121Aii** | HMP Gastrointestinal Tract |
| **Helicobacter_pylori_GAM201Ai** | HMP Gastrointestinal Tract |
| **Helicobacter_pylori_GAM210Bi** | HMP Gastrointestinal Tract |
| **Helicobacter_pylori_GAM231Ai** | HMP Gastrointestinal Tract |
| **Helicobacter_pylori_GAM239Bi** | HMP Gastrointestinal Tract |
| **Helicobacter_pylori_GAM244Ai** | HMP Gastrointestinal Tract |
| **Helicobacter_pylori_GAM245Ai** | HMP Gastrointestinal Tract |
| **Helicobacter_pylori_GAM246Ai** | HMP Gastrointestinal Tract |
| **Helicobacter_pylori_GAM249T** | HMP Gastrointestinal Tract |
| **Helicobacter_pylori_GAM250AFi** | HMP Gastrointestinal Tract |
| **Helicobacter_pylori_GAM250T** | HMP Gastrointestinal Tract |
| **Helicobacter_pylori_GAM252Bi** | HMP Gastrointestinal Tract |
| **Helicobacter_pylori_GAM252T** | HMP Gastrointestinal Tract |
| **Helicobacter_pylori_GAM254Ai** | HMP Gastrointestinal Tract |
| **Helicobacter_pylori_GAM260ASi** | HMP Gastrointestinal Tract |
| **Helicobacter_pylori_GAM260Bi** | HMP Gastrointestinal Tract |
| **Helicobacter_pylori_GAM260BSi** | HMP Gastrointestinal Tract |
| **Helicobacter_pylori_GAM263BFi** | HMP Gastrointestinal Tract |
| **Helicobacter_pylori_GAM264Ai** | HMP Gastrointestinal Tract |
| **Helicobacter_pylori_GAM265BSii** | HMP Gastrointestinal Tract |
| **Helicobacter_pylori_GAM268Bii** | HMP Gastrointestinal Tract |
| **Helicobacter_pylori_GAM270ASi** | HMP Gastrointestinal Tract |
| **Helicobacter_pylori_GAM42Ai** | HMP Gastrointestinal Tract |
| **Helicobacter_pylori_GAM71Ai** | HMP Gastrointestinal Tract |
| **Helicobacter_pylori_GAM80Ai** | HMP Gastrointestinal Tract |
| **Helicobacter_pylori_GAM83Bi** | HMP Gastrointestinal Tract |
| **Helicobacter_pylori_GAM83T** | HMP Gastrointestinal Tract |
| **Helicobacter_pylori_GAM93Bi** | HMP Gastrointestinal Tract |
| **Helicobacter_pylori_GAM96Ai** | HMP Gastrointestinal Tract |
| **Helicobacter_pylori_GAMchJs106B** | HMP Gastrointestinal Tract |
| **Helicobacter_pylori_GAMchJs114i** | HMP Gastrointestinal Tract |
| **Helicobacter_pylori_GAMchJs117Ai** | HMP Gastrointestinal Tract |
| **Helicobacter_pylori_GAMchJs124i** | HMP Gastrointestinal Tract |
| **Helicobacter_pylori_GAMchJs136i** | HMP Gastrointestinal Tract |
| **Helicobacter_pylori_HP116Bi** | HMP Gastrointestinal Tract |
| **Helicobacter_pylori_HP250AFii** | HMP Gastrointestinal Tract |
| **Helicobacter_pylori_HP250AFiii** | HMP Gastrointestinal Tract |
| **Helicobacter_pylori_HP250AFiV** | HMP Gastrointestinal Tract |
| **Helicobacter_pylori_HP250ASi** | HMP Gastrointestinal Tract |
| **Helicobacter_pylori_HP250ASii** | HMP Gastrointestinal Tract |
| **Helicobacter_pylori_HP250BFi** | HMP Gastrointestinal Tract |
| **Helicobacter_pylori_HP250BFii** | HMP Gastrointestinal Tract |
| **Helicobacter_pylori_HP250BFiii** | HMP Gastrointestinal Tract |
| **Helicobacter_pylori_HP250BFiV** | HMP Gastrointestinal Tract |
| **Helicobacter_pylori_HP250BSi** | HMP Gastrointestinal Tract |
| **Helicobacter_pylori_HP260AFi** | HMP Gastrointestinal Tract |
| **Helicobacter_pylori_HP260AFii** | HMP Gastrointestinal Tract |
| **Helicobacter_pylori_HP260ASii** | HMP Gastrointestinal Tract |
| **Helicobacter_pylori_HP260BFii** | HMP Gastrointestinal Tract |
| **Helicobacter_pylori_HP260Bi** | HMP Gastrointestinal Tract |
| **Helicobacter_winghamensis_ATCC_BAA-430** | HMP Gastrointestinal Tract |
| **Holdemania_filiformis_DSM_12042** | HMP Gastrointestinal Tract |
| **Intestinibacter_bartlettii_DSM_16795** | HMP Gastrointestinal Tract |
| **Klebsiella_pneumoniae_subsp._pneumoniae_WGLW3** | HMP Gastrointestinal Tract |
| **Klebsiella_pneumoniae_subsp._pneumoniae_WGLW5** | HMP Gastrointestinal Tract |
| **Klebsiella_sp._1_1_55** | HMP Gastrointestinal Tract |
| **Klebsiella_sp._4_1_44FAA** | HMP Gastrointestinal Tract |
| **Klebsiella_sp._MS_92-3** | HMP Gastrointestinal Tract |
| **Lachnospiraceae_bacterium_1_1_57FAA** | HMP Gastrointestinal Tract |
| **Lachnospiraceae_bacterium_1_4_56FAA** | HMP Gastrointestinal Tract |
| **Lachnospiraceae_bacterium_2_1_46FAA** | HMP Gastrointestinal Tract |
| **Lachnospiraceae_bacterium_2_1_58FAA** | HMP Gastrointestinal Tract |
| **Lachnospiraceae_bacterium_3_1_46FAA** | HMP Gastrointestinal Tract |
| **Lachnospiraceae_bacterium_3_1_57FAA_CT1** | HMP Gastrointestinal Tract |
| **Lachnospiraceae_bacterium_4_1_37FAA** | NCBI/PMID: 23363771 |
| **Lachnospiraceae_bacterium_5_1_57FAA** | HMP Gastrointestinal Tract |
| **Lachnospiraceae_bacterium_5_1_63FAA** | HMP Gastrointestinal Tract |
| **Lachnospiraceae_bacterium_6_1_37FAA** | HMP Gastrointestinal Tract |
| **Lachnospiraceae_bacterium_6_1_63FAA** | HMP Gastrointestinal Tract |
| **Lachnospiraceae_bacterium_7_1_58FAA** | HMP Gastrointestinal Tract |
| **Lachnospiraceae_bacterium_8_1_57FAA** | HMP Gastrointestinal Tract |
| **Lachnospiraceae_bacterium_9_1_43BFAA** | HMP Gastrointestinal Tract |
| **Lactobacillus_acidophilus_ATCC_4796** | HMP Gastrointestinal Tract |
| **Lactobacillus_amylolyticus_DSM_11664** | HMP Gastrointestinal Tract |
| **Lactobacillus_antri_DSM_16041** | HMP Gastrointestinal Tract |
| **Lactobacillus_brevis_subsp._gravesensis_ATCC_27305** | HMP Gastrointestinal Tract |
| **Lactobacillus_buchneri_ATCC_11577** | HMP Gastrointestinal Tract |
| **Lactobacillus_delbrueckii_subsp._lactis_DSM_20072** | HMP Gastrointestinal Tract |
| **Lactobacillus_fermentum_ATCC_14931** | HMP Gastrointestinal Tract |
| **Lactobacillus_fermentum_IFO_3956** | HMP Gastrointestinal Tract |
| **Lactobacillus_helveticus_DSM_20075** | HMP Gastrointestinal Tract |
| **Lactobacillus_hilgardii_ATCC_8290** | HMP Gastrointestinal Tract |
| **Lactobacillus_paracasei_subsp._paracasei_8700:2** | EMBL-EBI/PMID: 23363771 |
| **Lactobacillus_paracasei_subsp._paracasei_ATCC_25302** | HMP Gastrointestinal Tract |
| **Lactobacillus_plantarum_subsp._plantarum_ATCC_14917_=_JCM_1149_=** | HMP Gastrointestinal Tract |
| **Lactobacillus_reuteri_CF48-3A** | HMP Gastrointestinal Tract |
| **Lactobacillus_reuteri_JCM_1112** | HMP Gastrointestinal Tract |
| **Lactobacillus_reuteri_MM2-3** | HMP Gastrointestinal Tract |
| **Lactobacillus_reuteri_MM4-1A** | HMP Gastrointestinal Tract |
| **Lactobacillus_reuteri_SD2112** | HMP Gastrointestinal Tract |
| **Lactobacillus_rhamnosus_ATCC_21052** | HMP Gastrointestinal Tract |
| **Lactobacillus_rhamnosus_GG** | HMP Gastrointestinal Tract |
| **Lactobacillus_rhamnosus_LMS2-1** | HMP Gastrointestinal Tract |
| **Lactobacillus_ruminis_ATCC_25644** | HMP Gastrointestinal Tract |
| **Lactobacillus_salivarius_ATCC_11741** | NCBI/doi:10.1128/genomeA.01231-13 |
| **Lactobacillus_sp._7_1_47FAA** | HMP Gastrointestinal Tract |
| **Lactobacillus_ultunensis_DSM_16047** | HMP Gastrointestinal Tract |
| **Leuconostoc_mesenteroides_subsp._cremoris_ATCC_19254** | HMP Gastrointestinal Tract |
| **Listeria_grayi_DSM_20601** | HMP Gastrointestinal Tract |
| **Listeria_innocua_ATCC_33091** | HMP Gastrointestinal Tract |
| **Marvinbryantia_formatexigens_DSM_14469** | HMP Gastrointestinal Tract |
| **Megamonas_funiformis_YIT_11815** | HMP Gastrointestinal Tract |
| **Methanobrevibacter_smithii_DSM_2374** | HMP Gastrointestinal Tract |
| **Methanobrevibacter_smithii_DSM_2375** | HMP Gastrointestinal Tract |
| **Mitsuokella_multacida_DSM_20544** | HMP Gastrointestinal Tract |
| **Neisseria_macacae_ATCC_33926** | HMP Gastrointestinal Tract |
| **Odoribacter_laneus_YIT_12061** | HMP Gastrointestinal Tract |
| **Oxalobacter_formigenes_HOxBLS** | HMP Gastrointestinal Tract |
| **Oxalobacter_formigenes_OXCC13** | HMP Gastrointestinal Tract |
| **Paenibacillus_sp._HGF5** | HMP Gastrointestinal Tract |
| **Paenibacillus_sp._HGF7** | HMP Gastrointestinal Tract |
| **Paenibacillus_sp._HGH0039** | HMP Gastrointestinal Tract |
| **Paenisporosarcina_sp._HGH0030** | HMP Gastrointestinal Tract |
| **Parabacteroides_johnsonii_DSM_18315** | HMP Gastrointestinal Tract |
| **Parabacteroides_merdae_ATCC_43184** | HMP Gastrointestinal Tract |
| **Parabacteroides_sp._20_3** | HMP Gastrointestinal Tract |
| **Parabacteroides_sp._D13** | HMP Gastrointestinal Tract |
| **Paraprevotella_clara_YIT_11840** | HMP Gastrointestinal Tract |
| **Paraprevotella_xylaniphila_YIT_11841** | HMP Gastrointestinal Tract |
| **Parasutterella_excrementihominis_YIT_11859** | HMP Gastrointestinal Tract |
| **Parvimonas_micra_ATCC_33270** | HMP Gastrointestinal Tract |
| **Pediococcus_acidilactici_7_4** | HMP Gastrointestinal Tract |
| **Pediococcus_acidilactici_DSM_20284** | HMP Gastrointestinal Tract |
| **Peptoclostridium_difficile_70-100-2010** | HMP Gastrointestinal Tract |
| **Peptoclostridium_difficile_NAP07** | HMP Gastrointestinal Tract |
| **Peptoclostridium_difficile_NAP08** | HMP Gastrointestinal Tract |
| **Phascolarctobacterium_succinatutens_YIT_12067** | HMP Gastrointestinal Tract |
| **Prevotella_copri_DSM_18205** | HMP Gastrointestinal Tract |
| **Prevotella_oralis_HGA0225** | HMP Gastrointestinal Tract |
| **Prevotella_salivae_DSM_15606** | HMP Gastrointestinal Tract |
| **Prevotella_stercorea_DSM_18206** | HMP Gastrointestinal Tract |
| **Propionibacterium_sp._5_U_42AFAA** | HMP Gastrointestinal Tract |
| **Propionibacterium_sp._HGH0353** | HMP Gastrointestinal Tract |
| **Proteus_mirabilis_WGLW6** | HMP Gastrointestinal Tract |
| **Proteus_penneri_ATCC_35198** | HMP Gastrointestinal Tract |
| **Providencia_alcalifaciens_DSM_30120** | HMP Gastrointestinal Tract |
| **Providencia_rettgeri_DSM_1131** | HMP Gastrointestinal Tract |
| **Providencia_rustigianii_DSM_4541** | HMP Gastrointestinal Tract |
| **Providencia_stuartii_ATCC_25827** | HMP Gastrointestinal Tract |
| **Pseudoflavonifractor_capillosus_ATCC_29799** | HMP Gastrointestinal Tract |
| **Pseudomonas_sp._2_1_26** | HMP Gastrointestinal Tract |
| **Ralstonia_sp._5_2_56FAA** | HMP Gastrointestinal Tract |
| **Ralstonia_sp._5_7_47FAA** | HMP Gastrointestinal Tract |
| **Roseburia_intestinalis_L1-82** | HMP Gastrointestinal Tract |
| **Roseburia_intestinalis_XB6B4** | HMP Gastrointestinal Tract |
| **Roseburia_inulinivorans_DSM_16841** | HMP Gastrointestinal Tract |
| **Ruminococcaceae_bacterium_D16** | HMP Gastrointestinal Tract |
| **Ruminococcus_bromii_L2-63** | HMP Gastrointestinal Tract |
| **Ruminococcus_champanellensis_18P13_=_JCM_17042** | HMP Gastrointestinal Tract |
| **Ruminococcus_gnavus_ATCC_29149** | HMP Gastrointestinal Tract |
| **Ruminococcus_lactaris_ATCC_29176** | HMP Gastrointestinal Tract |
| **Ruminococcus_obeum_A2-162** | HMP Gastrointestinal Tract |
| **Ruminococcus_obeum_ATCC_29174** | HMP Gastrointestinal Tract |
| **Ruminococcus_sp._5_1_39BFAA** | HMP Gastrointestinal Tract |
| **Ruminococcus_torques_ATCC_27756** | HMP Gastrointestinal Tract |
| **Ruminococcus_torques_L2-14** | HMP Gastrointestinal Tract |
| **Slackia_piriformis_YIT_12062** | HMP Gastrointestinal Tract |
| **Staphylococcus_sp._HGB0015** | HMP Gastrointestinal Tract |
| **Streptococcus_anginosus_1_2_62CV** | HMP Gastrointestinal Tract |
| **Streptococcus_equinus_ATCC_9812** | HMP Gastrointestinal Tract |
| **Streptococcus_infantarius_subsp._infantarius_ATCC_BAA-102** | HMP Gastrointestinal Tract |
| **Streptococcus_sp._2_1_36FAA** | HMP Gastrointestinal Tract |
| **Streptococcus_sp._HPH0090** | HMP Gastrointestinal Tract |
| **Streptomyces_sp._HGB0020** | HMP Gastrointestinal Tract |
| **Streptomyces_sp._HPH0547** | HMP Gastrointestinal Tract |
| **Subdoligranulum_sp._4_3_54A2FAA** | HMP Gastrointestinal Tract |
| **Subdoligranulum_variabile_DSM_15176** | HMP Gastrointestinal Tract |
| **Succinatimonas_hippei_YIT_12066** | HMP Gastrointestinal Tract |
| **Sutterella_parvirubra_YIT_11816** | HMP Gastrointestinal Tract |
| **Sutterella_wadsworthensis_2_1_59BFAA** | HMP Gastrointestinal Tract |
| **Sutterella_wadsworthensis_3_1_45B** | HMP Gastrointestinal Tract |
| **Sutterella_wadsworthensis_HGA0223** | HMP Gastrointestinal Tract |
| **Synergistes_sp._3_1_syn1** | HMP Gastrointestinal Tract |
| **Tannerella_sp._6_1_58FAA_CT1** | HMP Gastrointestinal Tract |
| **Turicibacter_sanguinis_PC909** | HMP Gastrointestinal Tract |
| **Turicibacter_sp._HGF1** | HMP Gastrointestinal Tract |
| **Tyzzerella_nexilis_DSM_1787** | HMP Gastrointestinal Tract |
| **Veillonella_sp._3_1_44** | HMP Gastrointestinal Tract |
| **Veillonella_sp._6_1_27** | HMP Gastrointestinal Tract |
| **Veillonella_sp._HPA0037** | HMP Gastrointestinal Tract |
| **Weissella_paramesenteroides_ATCC_33313** | HMP Gastrointestinal Tract |
| **Yokenella_regensburgei_ATCC_43003** | HMP Gastrointestinal Tract |
| **Methanobrevibacter_smithii_ATCC_35061** | NCBI/PMID: 23363771 |
| **Parabacteroides_distasonis_ATCC_8503** | NCBI/PMID: 23363771 |

**Table S5.** Number of molecules used as blind set for each EC class RF models.

| **Major EC class** | **Number of molecules** |
| --- | --- |
| **EC1** | 61 |
| **EC2** | 45 |
| **EC3** | 30 |
| **EC4** | 15 |
| **EC5** | 5 |
| **EC6** | 6 |

**Figure S1. a)** Distribution of substrates among six EC classes, **b-g)** Distribution of substrates among EC subclasses**.**

**a)**

**
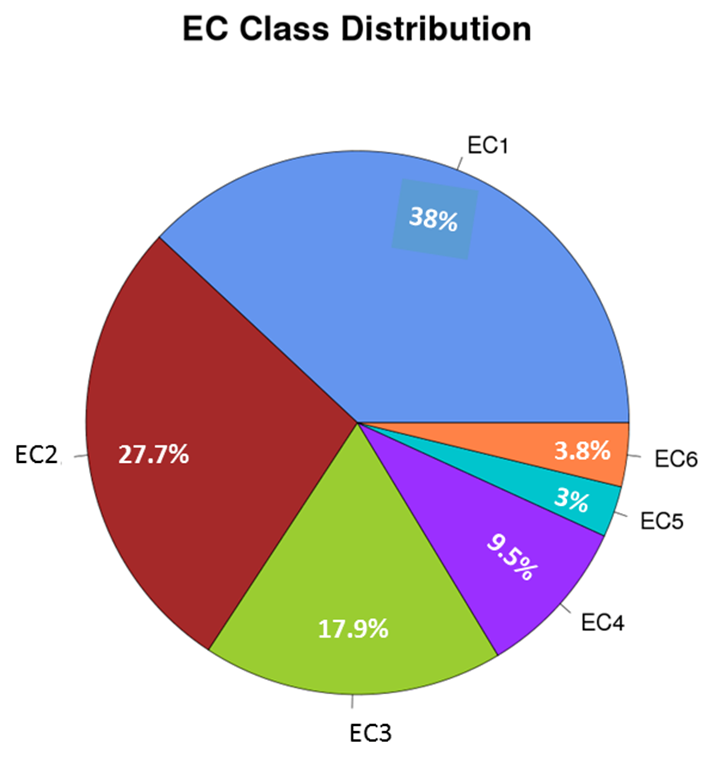
**

**b)**

**
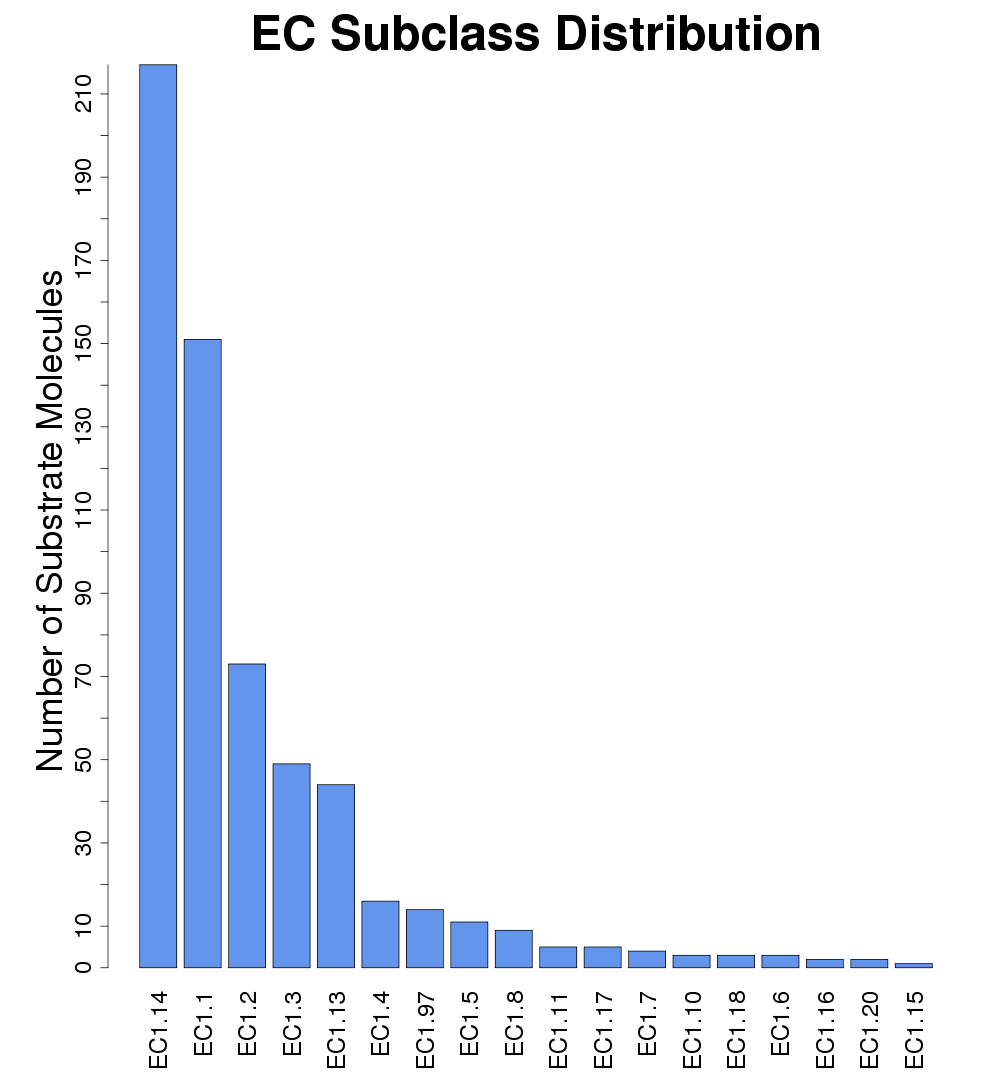
**

**c)**

**
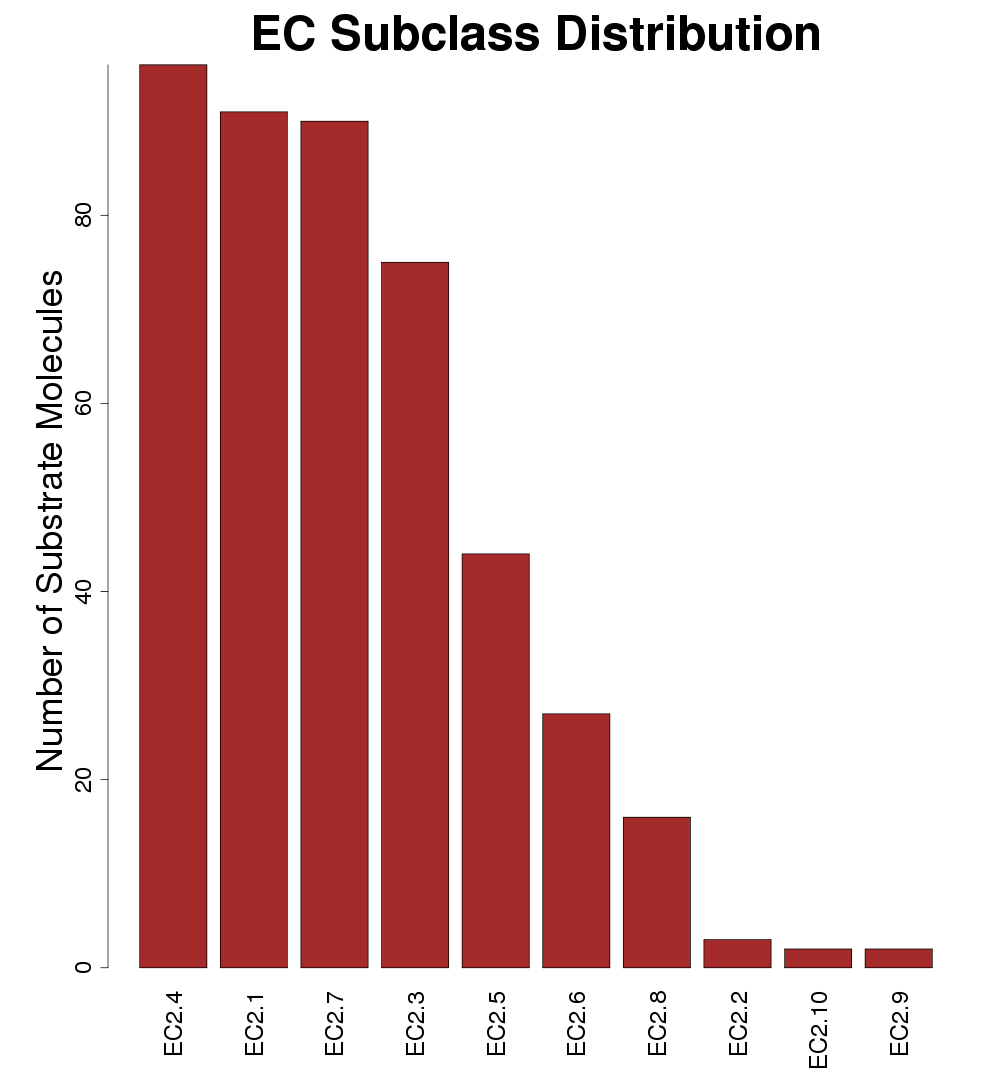
**

**d)**

**
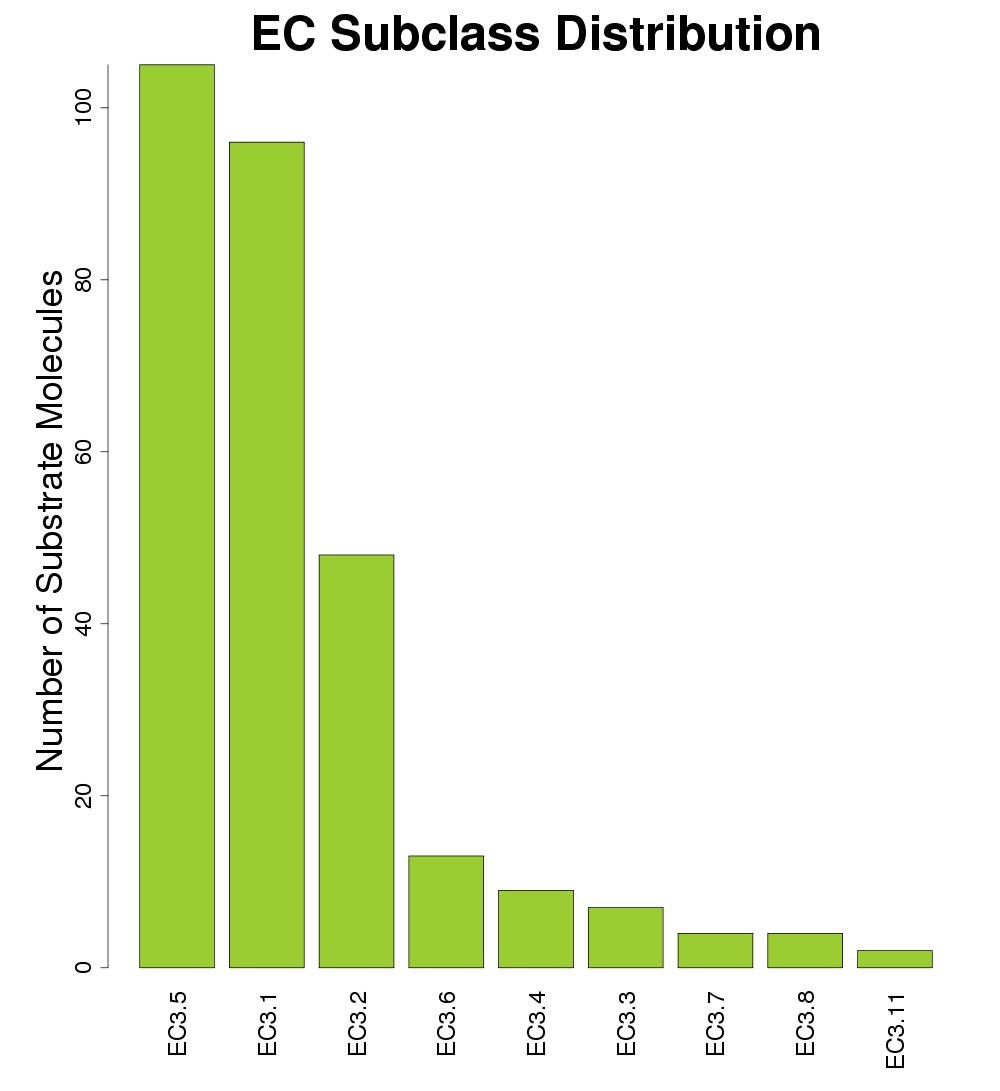
**

**e)**

**
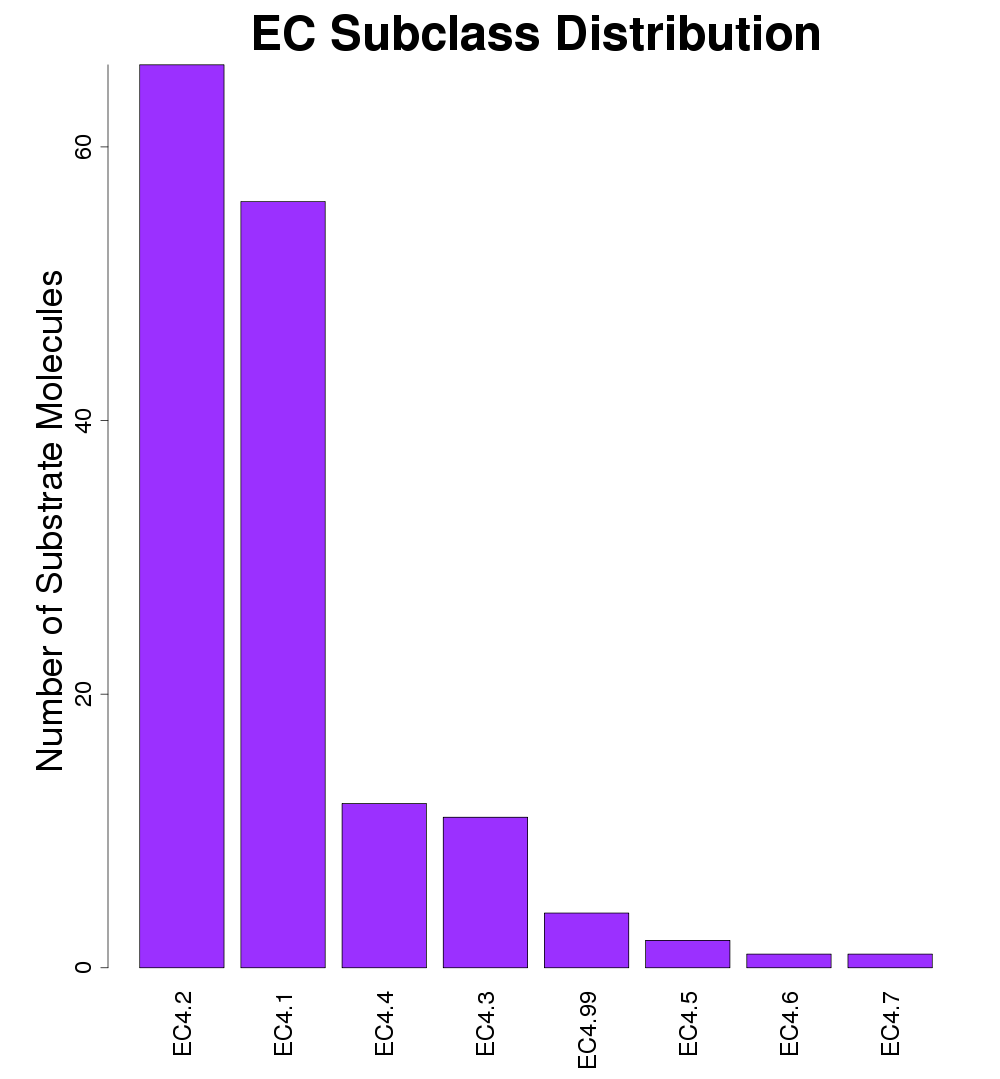
**

**f)**

**
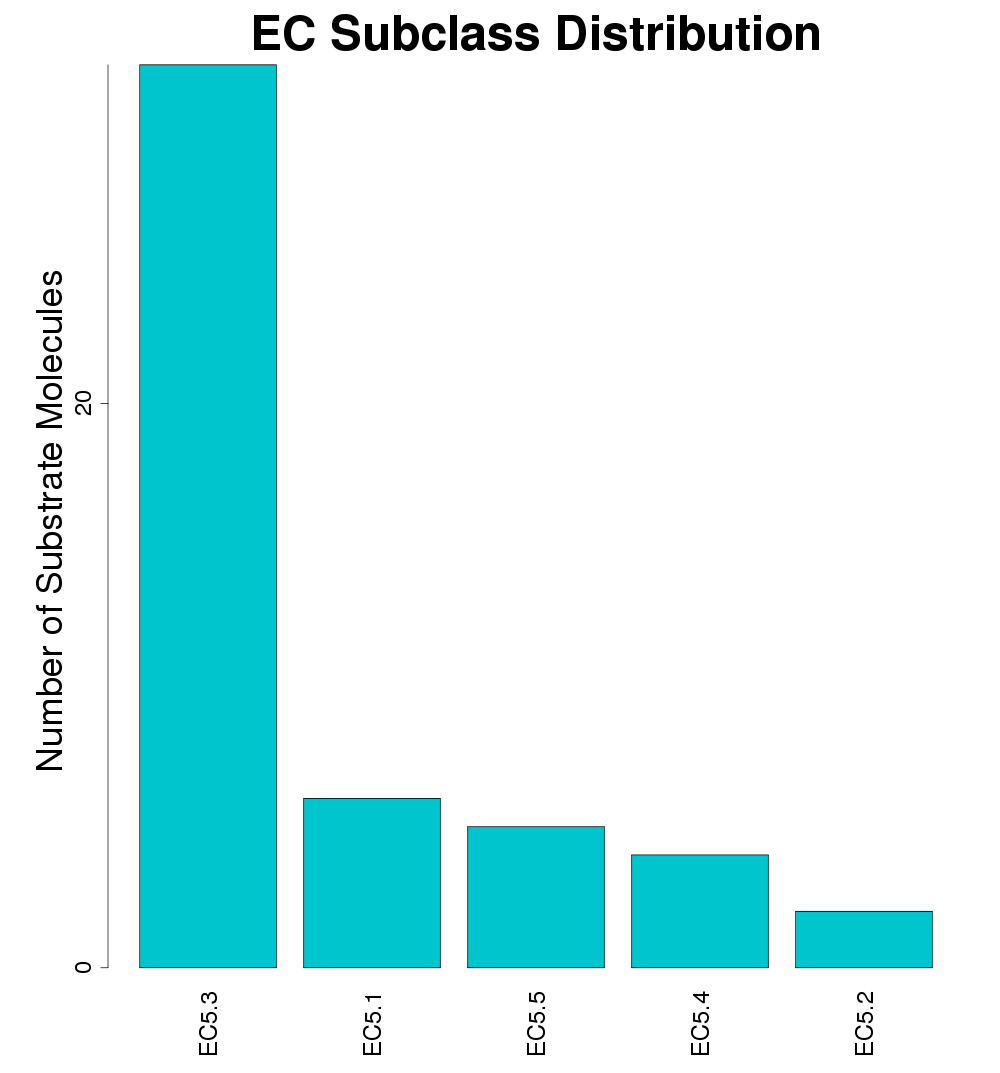
**

**g)**

**
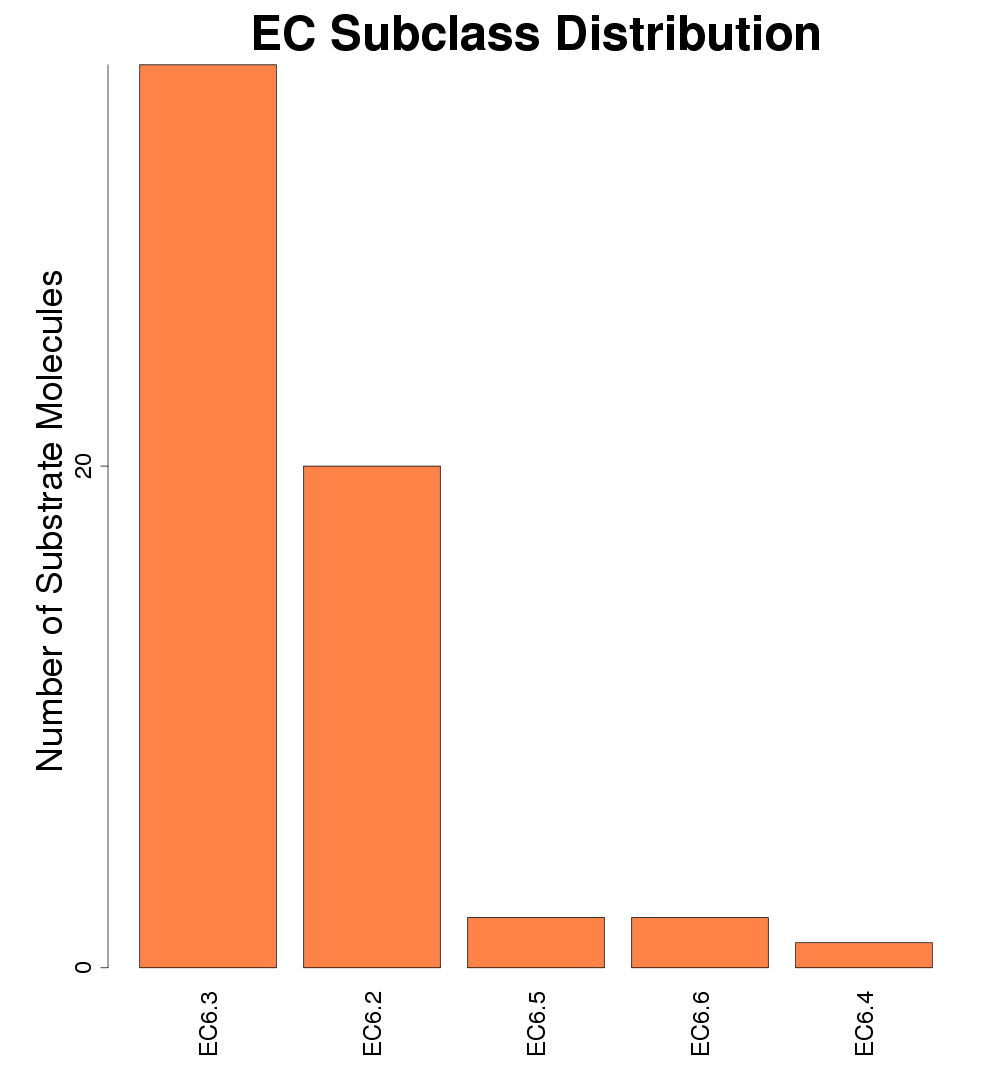
**

**Figure S2. a)** Variance of the Principal Components among the six EC classes, **b)** Variance of the Principal Components among EC subclasses of six EC classes.

**
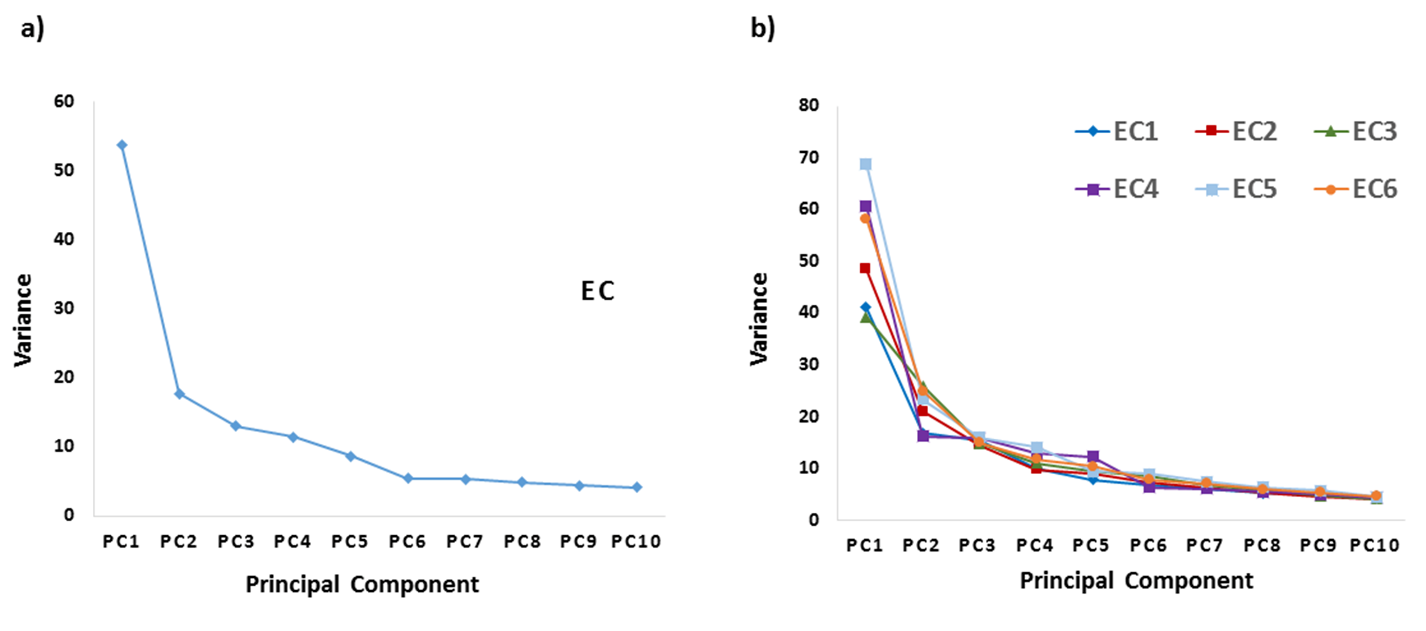
**

**Figure S3.** Principal Component Analysis showing the distribution of substrate molecules in the EC subclasses.

**a)**

**
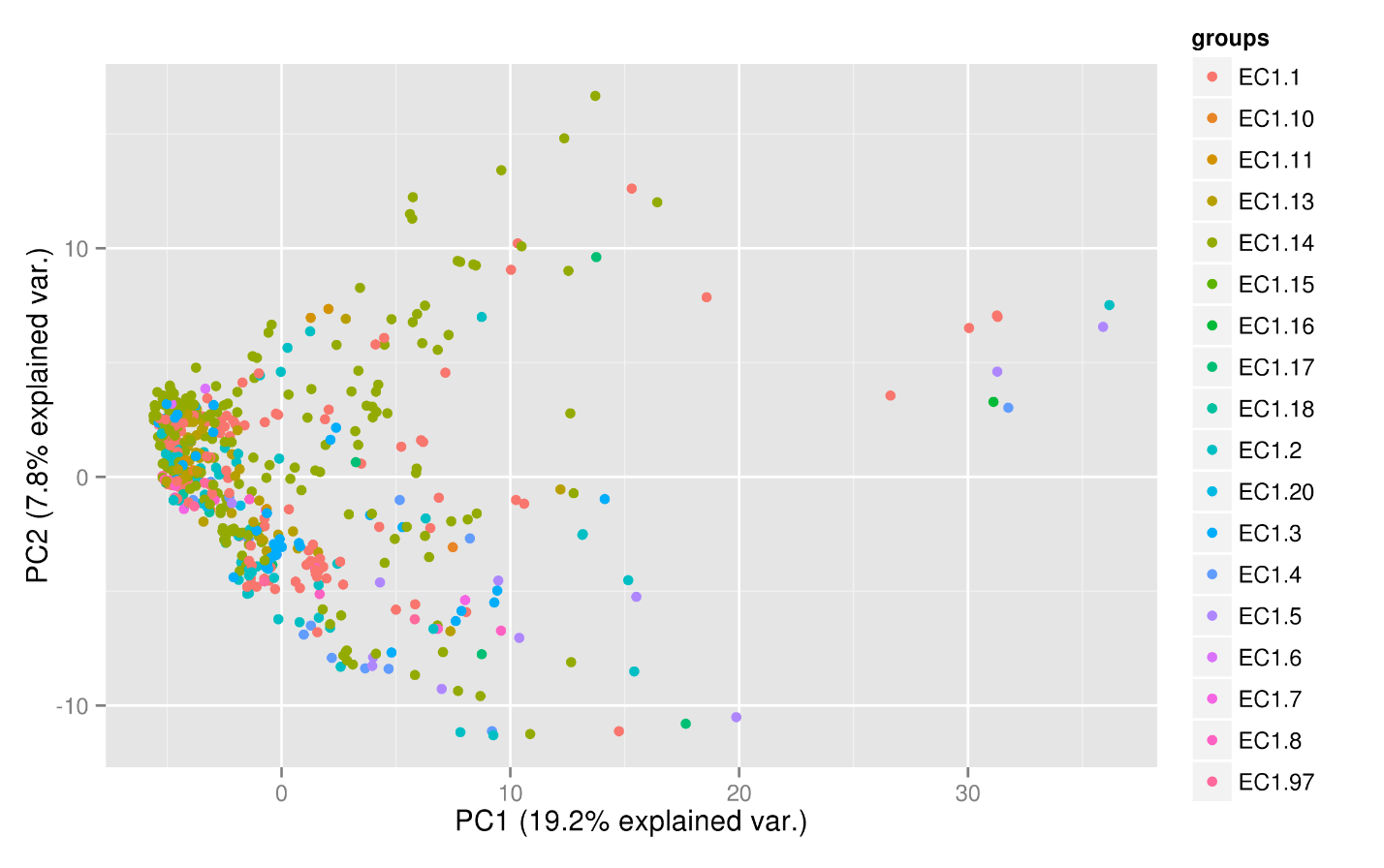
**

**b)**

**
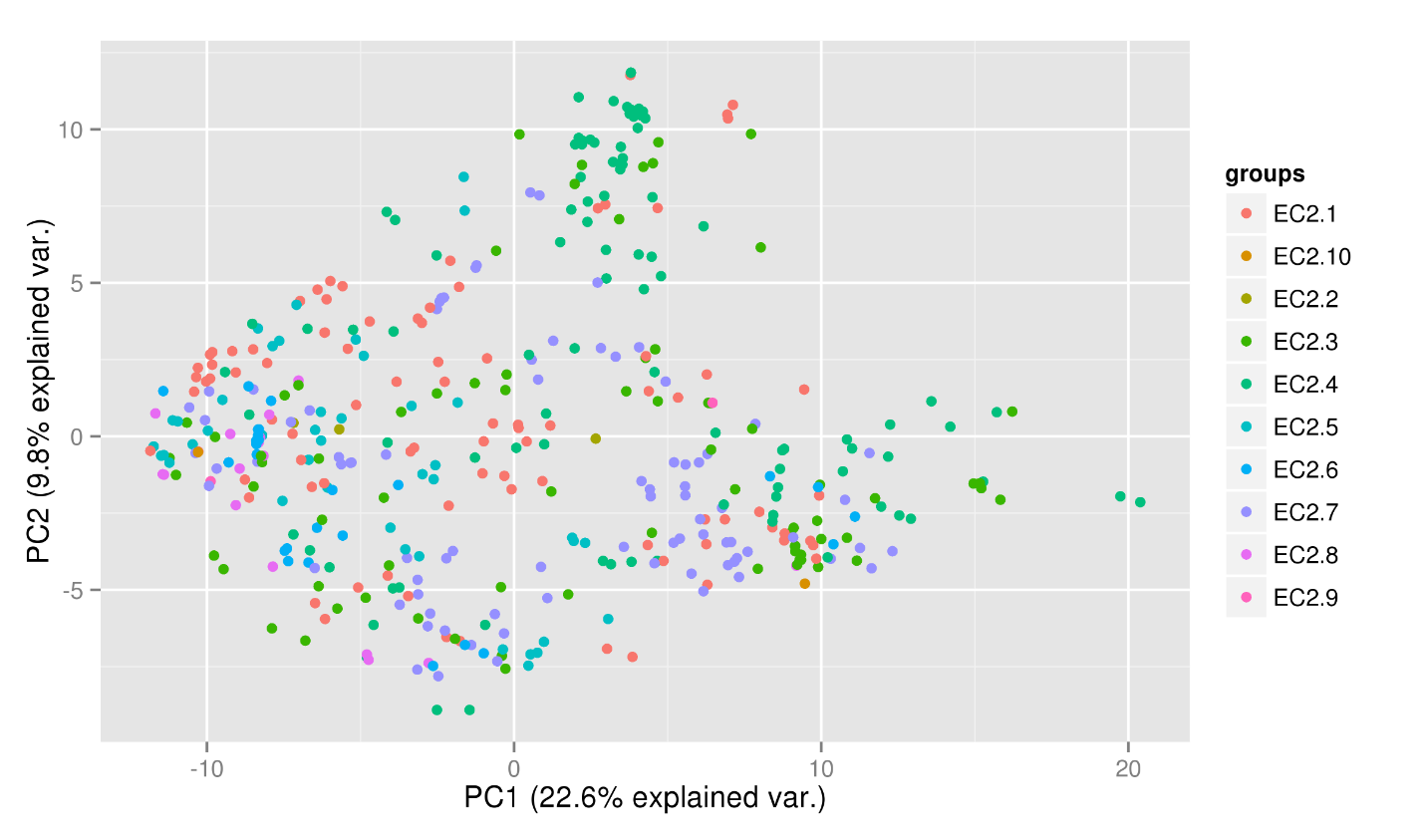
**

**c)**

**
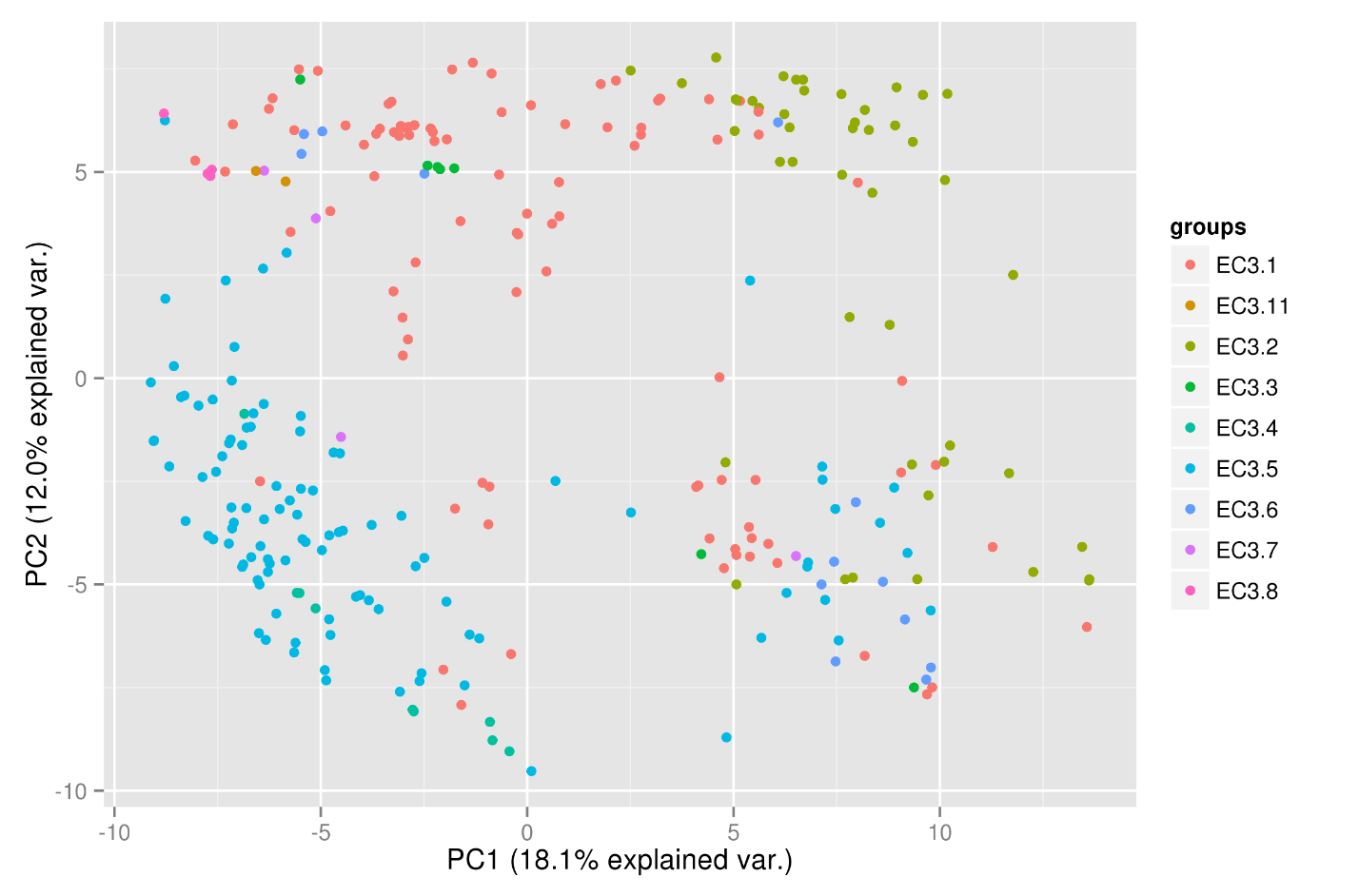
**

**d)**

**
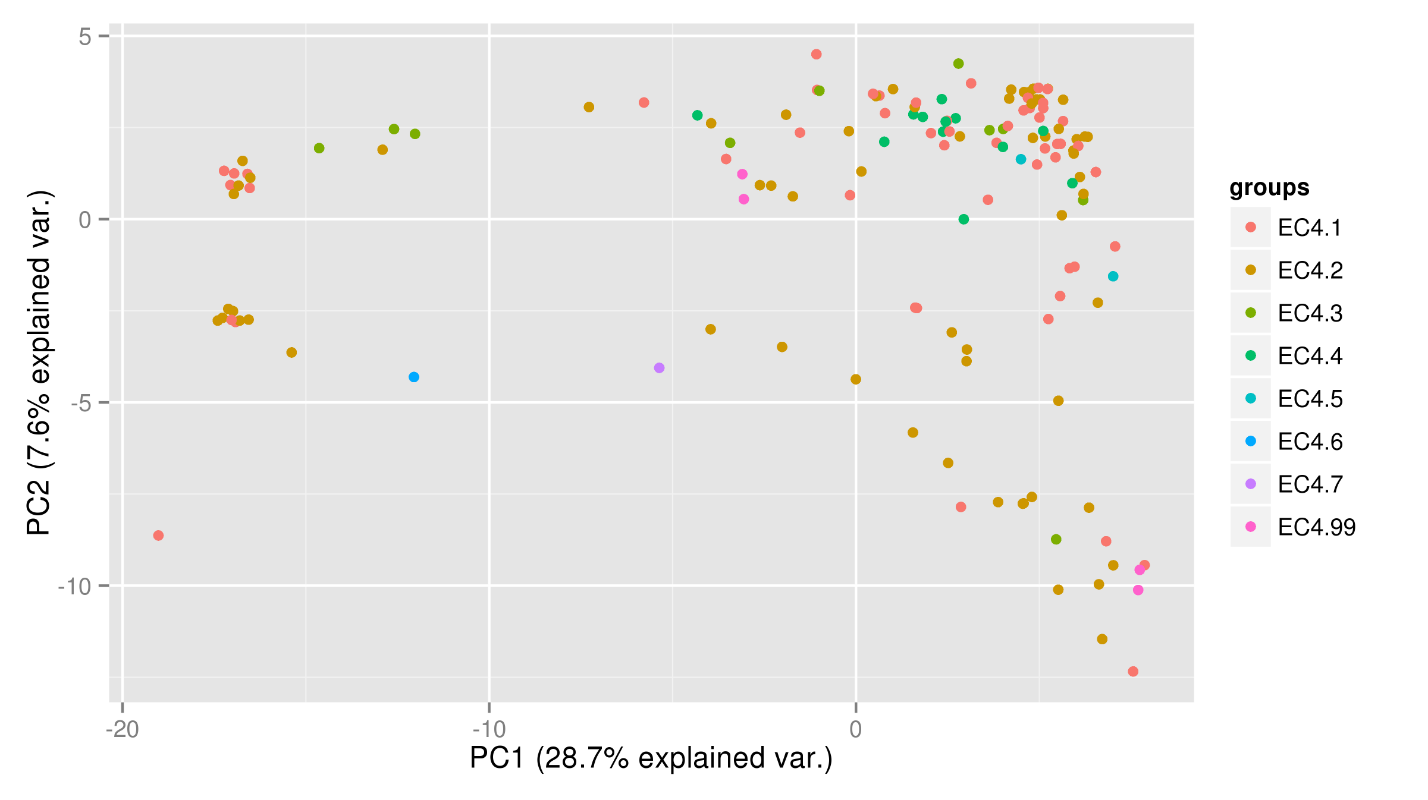
**

**e)**

**
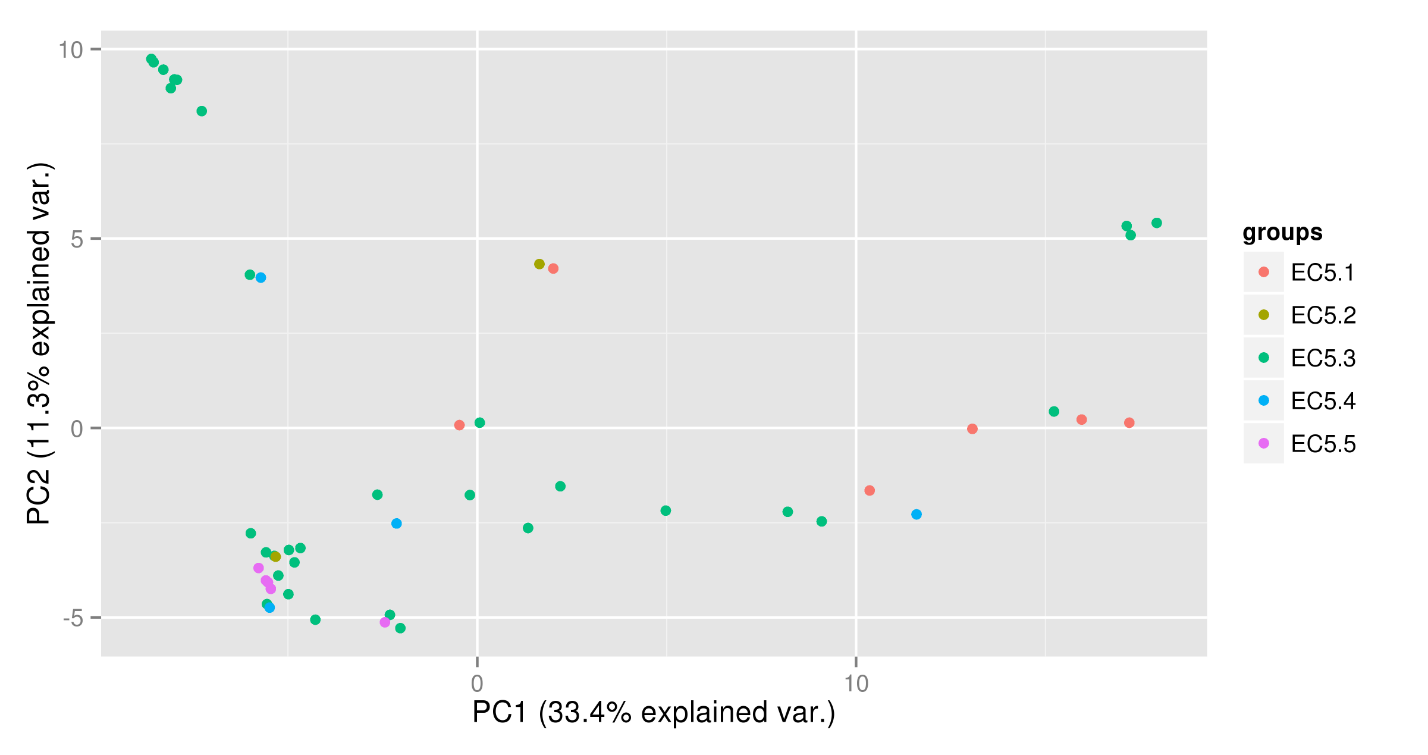
**

**f)**

**
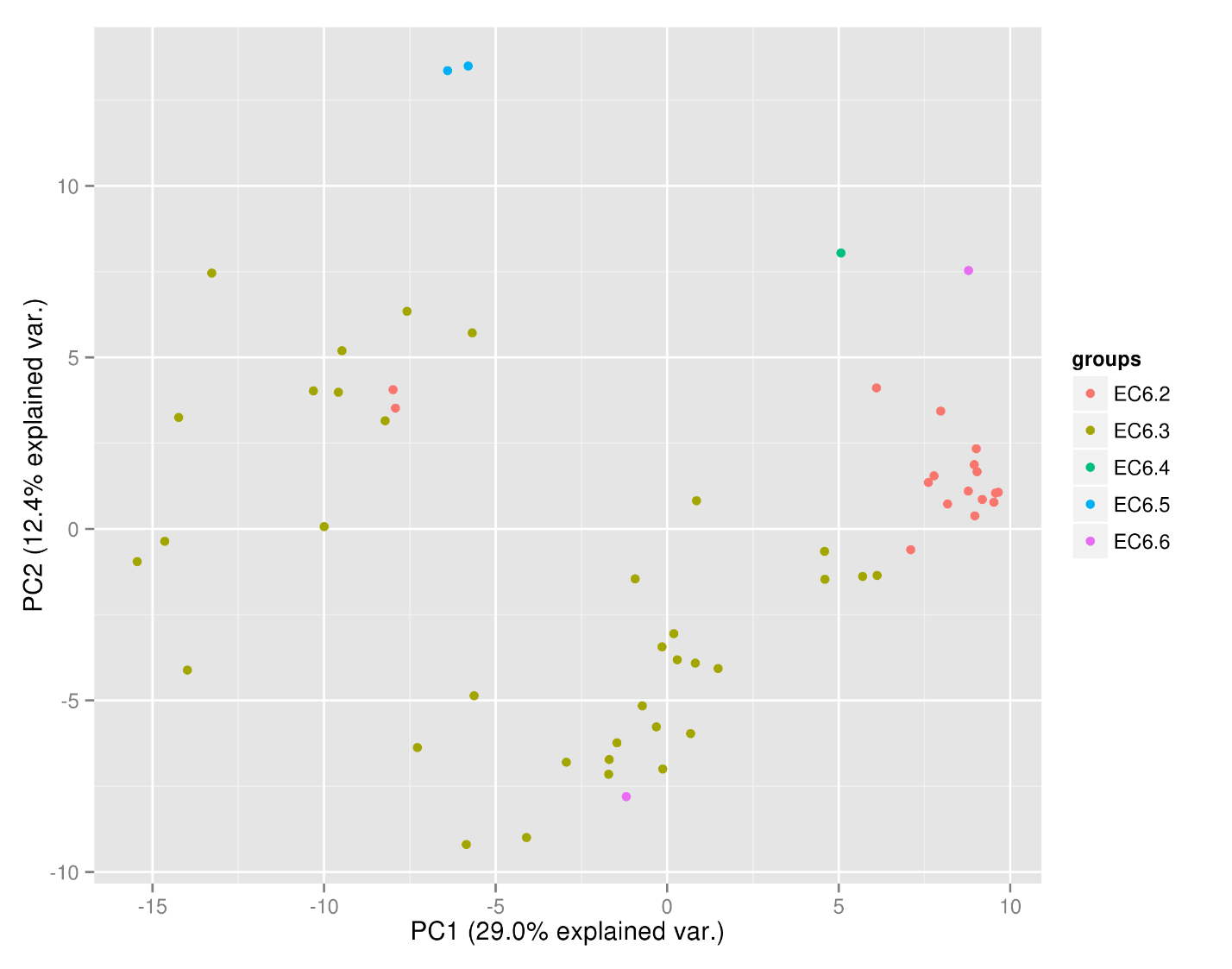
**

**Figure S4.** Optimization of parameters (mtry, fingerprint and dataset) to construct the final RF model for classification into EC subclasses.


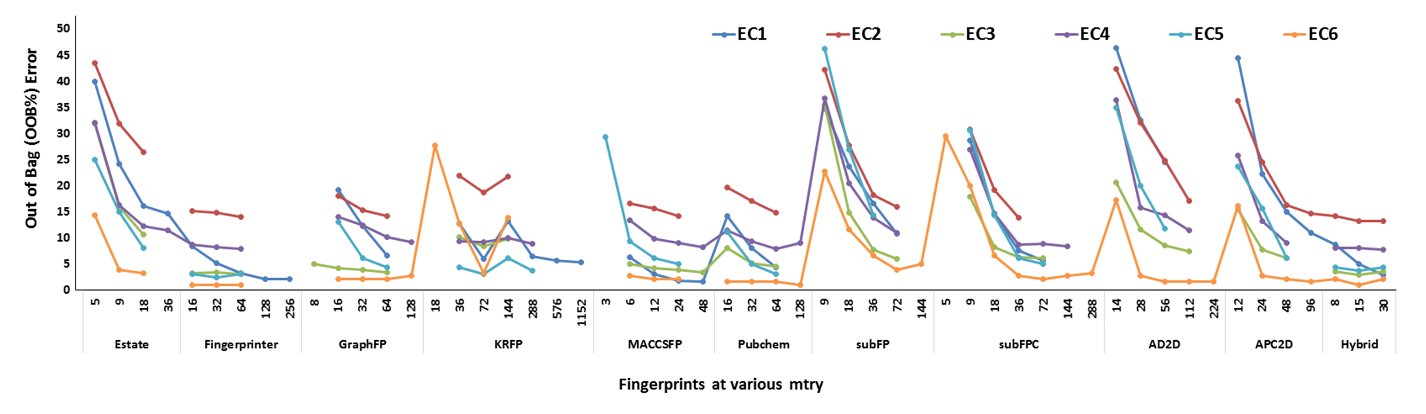


**Figure S5.** Construction of hybrid fingerprint using 10 standard fingerprints


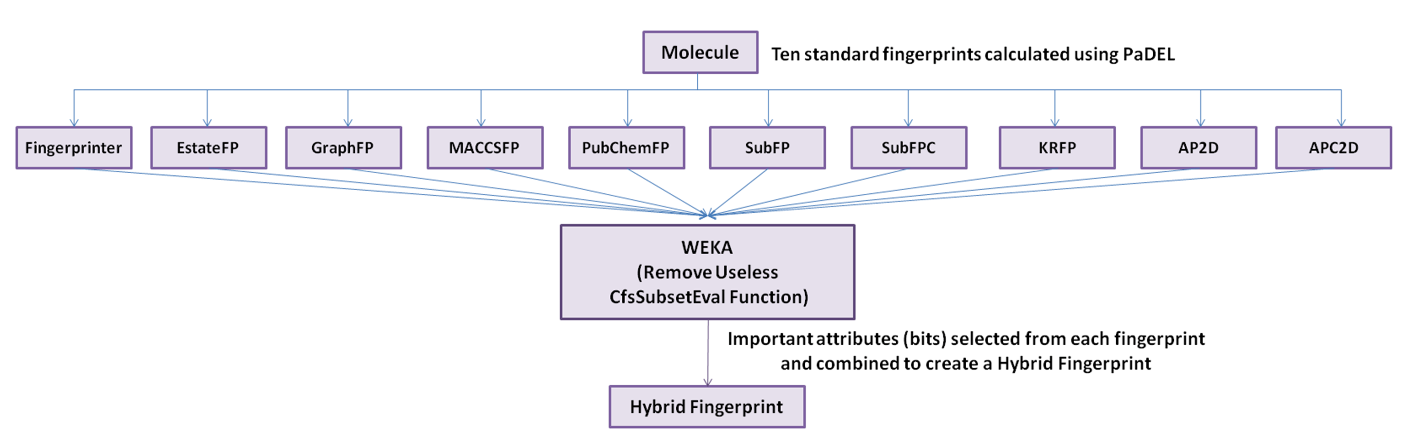

Supplement: Supplementary file 1 — Supplementary Information [file 41598_2017_10203_MOESM1_ESM.doc]
